# A Double-Stranded Aptamer for Highly Sensitive Fluorescent Detection of Glutathione S-Transferases

**DOI:** 10.3390/bios14100476

**Published:** 2024-10-03

**Authors:** Wei Cui, Suping Li, Jiahao Zeng, Chen Li, Zhaofeng Li, Xiaohong Wen, Suxia Bao, Yang Mei, Xiangxian Meng, Qiuping Guo

**Affiliations:** 1 College of Biology, State Key Laboratory of Chemo/Biosensing and Chemometrics, College of Chemistry and Chemical Engineering, Hunan University, Key Laboratory for Bio-Nanotechnology and Molecule Engineering of Hunan Province, Changsha 410082, China; cuiwei@hnu.edu.cn (W.C.);; 2 School of Biomedical Sciences, Hunan University, Changsha 410082, China; 3 College of Biology, Hunan University, Changsha 410082, China

**Keywords:** aptamer, glutathione S-transferase (GST), SELEX, enzyme detection

## Abstract

Aptamer-based biosensors have been widely constructed and applied to detect diverse targets. Glutathione S-transferase (GST), a pivotal phase II metabolic enzyme, plays a critical role in biotransformation in vivo, and aberrant GST expression is associated with various health risks. Herein, aptamers targeting GST were systematically selected from a randomized single-stranded DNA (ssDNA) library of 79 nucleotides (nt) using a biotinylated GST-immobilized streptavidin agarose (SA) bead SELEX technology. Following rigorous screening across eight rounds, four aptamers with strikingly similar secondary structures emerged. Among these, Seq3 exhibited the highest affinity towards GST and was selected for further optimization. A semi-rational post-SELEX truncation strategy was then employed based on base composition analysis, secondary structure analysis and affinity assessment. This strategy enabled the systematic removal of redundant nucleotides in Seq3 without compromising its affinity, ultimately yielding a truncated aptamer, Seq3-3, which retains its specificity with a compact 39nt length. Building upon Seq3-3, a double-stranded fluorescent aptamer probe was ingeniously designed for the in vitro detection of GST. The detection mechanism hinges on the competitive displacement of the complementary chain from the probe, mediated by the target protein, leading to the separation of the antisense oligonucleotide from the double-stranded complex. This process triggers the restoration of the fluorescence signal, enabling sensitive detection, and the probe exhibits excellent response within a linear range of GST activity ranging from 0 to 1500 U/L. The results show that not only an efficient strategy for screening robust and practicable aptamers but also an ultrahighly sensitive detection platform for GST was established.

## 1. Introduction

Glutathione S-transferase (GST), a multifaceted family of phase II metabolic enzymes, is ubiquitously distributed across mitochondria, microsomes, and cytoplasm, playing pivotal roles in the detoxification processes within cells [1]. Originating from its discovery in rat livers in the early 1960s, GST is now known to pervade a wide array of living organisms [2]. Its principal cytoprotective mechanism involves catalyzing the conjugation of glutathione (GSH) with metabolites from cytochrome P450, thereby facilitating the protective formation of GSH conjugates. In addition to detoxification [3,4], GST also has functions in cell signal transduction, post-translational modification and cell drug resistance [5,6]. The up-regulated expression of GST can be used as a potential biomarker in the occurrence of various diseases such as cell injury, tumorgenesis and tumor drug resistance [7,8]. Therefore, it is important to achieve a highly sensitive and highly specific quantitative determination of GST. Currently, the method of enzyme activity detection of GST is mainly based on fluorescent substrates [9], chemiluminescence [10], radioisotope labeling [11], and enzyme-linked immunoassay [12]. Each method outlined above bears distinct advantages and limitations, necessitating the selection of the most suitable approach tailored to specific research objectives and experimental contexts. At present, GST biosensors have been able to detect and monitor GST activity in biological samples with specific binding capabilities [13,14] and have good biocompatibility for live cell imaging and in vivo applications [15]. Some designs take into account real-time monitoring of GST activity changes and quantitative analysis, which is of great significance for the study of dynamic physiological processes and the mechanism of disease occurrence and development [16].

As biotechnology advances, biosensors, equipped with high-specificity recognition elements and efficient signal amplification mechanisms, have emerged as a powerful tool, offering precise and swift detection capabilities for a wide array of targets. Presently, GST fluorescent probes, though reliant on GST-catalyzed nucleophilic substitution reactions involving GSH, face challenges in cellular environments rich in nucleophilic species, which can lead to interference. In addition, the diversity and representativeness of constructed libraries will directly affect the screening results, and the screening efficiency and success rate are also issues to be considered in technological innovation in order to identify a single sequence with high affinity and specificity. Moreover, the functions of cellular GST are generally overlooked due to the lack of suitable luminescence probes, and applications of aptamers may be limited by factors such as their stability, solubility, and toxicity in vivo. Thus, there is an urgent need for the development of novel, effective molecular probes specifically targeted at GST to enhance detection precision and efficiency.

Aptamers, single-stranded oligonucleotide molecules evolved by SELEX (systematic evolution of ligands by exponential enrichment), can selectively and specifically bind to their targets and have become attractive alternatives to traditional antibodies [17,18]. Since the inception of SELEX technology, the evolution and application of aptamer-based biosensors have seen a surge in diverse fields [19,20,21]. In this paper, we unveil Seq3-3, a DNA aptamer acting as a nucleic acid mimic with a specific affinity for GST. Our selection process diverges from conventional protein filtration through cellulose nitrate membranes, which often leads to false positives due to non-specific adsorption and complex screening logistics. Instead, aptamers were meticulously selected from a randomized single-stranded DNA (ssDNA) pool, leveraging the distinctive enzyme–substrate interactions between GST tags and GSH on sepharose beads (SA beads). The separation of free ssDNA and protein-bound ssDNA by centrifugation significantly improves the screening efficiency. We have engineered a distinctive double-stranded fluorescent aptamer probe in detoxification processes and a biomarker in various diseases. This probe not only stands out for its precision and reliability but also offers significant practical advantages over existing fluorescent probes and detection technologies. Our probe’s unique structure and fluorescent properties enable it to bind specifically to GST with high affinity and sensitivity, outperforming conventional detection methods in terms of specificity and ease of use.

## 2. Experimental Section

### 2.1. Chemicals and Materials

All DNA oligonucleotides (listed in Table 1) were synthesized and HPLC-purified by Sangon Biotechnology Co., Ltd. (Shanghai, China). All the chemicals were used as received without further purification. Adenosine triphosphate (ATP), cytidine triphosphate (CTP), guanosine triphosphate (GTP), and uridine triphosphate (UTP) were purchased from Sangon Biotechnology Co., Ltd. (Shanghai, China). Dulbecco’s phosphate-buffered saline (DPBS) and bovine serum albumin (BSA) were obtained from Sigma-Aldrich (St. Louis, MO, USA). The loading buffer and DNA marker were ordered from TaKaRa Bio Inc. (Dalian, China). SYBR Gold was purchased from Invitrogen Company, Ltd. (Carlsbad, CA, USA). Yeast tRNA, glucose, and MgCl_2_∙6H_2_O were purchased from Sinopharm Chemical Reagent Co., Ltd. (Shanghai, China). The binding buffer was prepared by adding 1 mg/mL BSA and 0.1 mg/mL yeast tRNA into DPBS containing 4.5 mg/mL glucose and 5 mM MgCl_2_∙6H_2_O. All solutions were prepared and diluted using ultrapure water (>18.2 MΩ cm, 25 °C), which was provided by a Milli-Q water purification system (Millipore, Burlington, MA, USA).

### 2.2. Screening Condition

In order to ensure the affinity of ssDNA to the target, we chose to incubate in a binding buffer (BB, 2 mM MgCl_2_, 5 mM KCl, 120 mM NaCl, 20 mM HEPES, pH 7.4) and eluate it with a washing buffer (WB, 0.05% Tween 20, 1 × BB). With the progress of screening, the position screening time was gradually shortened from 40 min to 30 min, while the control screening time was gradually increased from 10 min to 30 min. At the same time, the washing conditions were becoming more and more strict, and the volume of liquid, the number, and the time were continuously increased with the SELEX. Specific operations are as follows:

(1) Library pre-treatment: The initial library, containing 4 nanomoles, is first subjected to centrifugation at 12,000 revolutions per minute (rpm) at a temperature of 4 °C for 5 min. Following this, 400 microliters (µL) of combined buffer BB is added to dissolve the library. The solution is then heated to 95 °C for 5 min in a water bath, after which it is promptly transferred to ice to achieve rapid cooling.
(2) Pre-incubation: 1 mL of BB and 5 µL of a 50 µM solution of unrelated sequences to separate centrifuge tubes containing target and control proteins, respectively. Pre-incubate the mixtures at room temperature for 5 min to allow for initial interactions. Following incubation, use a centrifuge to remove the supernatant. Proceed to wash the proteins three times with 1 mL of WB, ensuring thorough removal of unbound components.
(3) Positive and control screening: Add the pre-conditioned library to the pre-incubated target protein, and the third round begins to introduce control screening. Incubate at 37 °C and shake well at 80 r/min so that it is fully incubated. Centrifuge and retain the supernatant.
(4) Elution and separation: Post incubation, perform a centrifugation step to isolate the microbeads, discarding the supernatant. Initiate washing with WB, progressively increasing the volume from 400 µL to 800 µL while extending the washing time from 30s to 60s and boosting the number of elution cycles from 2 to 4. The ssDNA with weak binding ability and non-specific binding ability is eluted, and the ssDNA with strong binding ability is retained.
(5) Collection of libraries post incubation: ① Add 800 µL of sterile water to the microbeads (500 µL in the first round). ② Place the centrifuge tube in a 95 °C water bath for 10 min to induce protein denaturation and facilitate the release of ssDNA from their complexes. ③ Rapidly transfer the tube to ice for 5 min to cool down, stabilizing the released ssDNA. The mixture was then centrifuged, and the supernatant containing the target sequence was collected, labeled as “Round n”, and cryopreserved.

### 2.3. PCR Programming

First, we used PCR (Mastercycler pro, Eppendorf, Germany) to automatically generate 10 annealing temperatures of 55.2 °C, 55.8 °C, 56.7 °C, 57.8 °C, 59.1 °C, 60.4 °C, 61.7 °C, 62.9 °C, 63.9 °C, and 64.6 °C. The optimized annealing temperature was obtained by 3% agarose gel electrophoresis. As shown in Figure S1 (Supplementary Materials), the brightness of the bands indicated the highest PCR yield, and the non-specificity of the strip became weaker with the increase in annealing temperature. Therefore, the temperature corresponding to lane 7 (60.4 °C) was selected as the optimal annealing temperature. Secondly, in order to ensure that the number of libraries obtained in each round of screening is sufficient for the next round of screening input while avoiding the generation of non-specific products, we optimized the number of cycles for each round of PCR amplification. The optimal number of cycles from 1 to 9 and the verification results are shown in Figure S2 (Supplementary Materials). The selection criteria followed the principle of clear, single, and bright bands, and in order to ensure the specificity of the product, a certain yield could be sacrificed and fewer rounds were selected as the optimal number of cycles.

### 2.4. Characterization of the Screening Process

The samples were imaged using laser scanning confocal microscopy (LSCM, Nikon, Japan) to monitor the library enrichment during the screening process. Each round of libraries and target protein (50 μL) were prepared into a 200 μL system and incubated at 37 °C for 40 min, and the final concentration of the library was 500 nM. After incubation and washing three times with WB, centrifuge and discard the supernatant. Finally, 80 μL BB was added and suspended, and 20 μL was added to the slide after mixing. The combination between the library and the target was analyzed by laser confocal detection. The AF488 fluorescence signal was collected with a 100× oil immersion objective (fluorescence channel: EX 488 nm, EM 500–550 nm band-pass).

### 2.5. Determination of Dissociation Equilibrium Constant

The sequence with strong library and binding ability was prepared into a 200 μL system with the target protein (50 μL) and incubated at 37 °C for 40 min. The final concentration of the sequence was 5, 10, 25, 50, 100, 250, 500, and 1000 nM concentration gradient, respectively. Then the supernatant was centrifuged and washed three times with WB; centrifuge the supernatant and add 80 μL of BB. The samples were imaged using laser scanning confocal microscopy (LSCM, Nikon, Japan), and Image J analyzed the average fluorescence intensity of microbeads in the image. The average fluorescence intensity was produced by subtracting the non-specific adsorption of microbeads. The dissociation equilibrium constant (Kd) of the aptamer was simulated using the model Y = B_max_X/(Kd + X); the horizontal coordinate is the concentration of the aptamer, and the longitudinal is the difference in the mean fluorescence intensity.

### 2.6. Statistical Analysis

Initially, we undertook the preprocessing of the raw data, specifically applying data normalization techniques to standardize datasets of varying scales and numerical ranges, ensuring they align with the characteristics of a standard normal distribution. This normalization step is foundational to enhancing the performance and accuracy of algorithms, improving numerical stability, and mitigating potential biases that could arise from disparate data magnitudes. Using the normalized results to plot, GraphPad Prism 9.0′s histograms and Sigmaplot’s curves were used to visually observe the experimental results. All study data are included in this article or the Supplementary Materials.

## 3. Results and Discussion

### 3.1. Selection of DNA Aptamer against GST Protein

**Figure 1:**
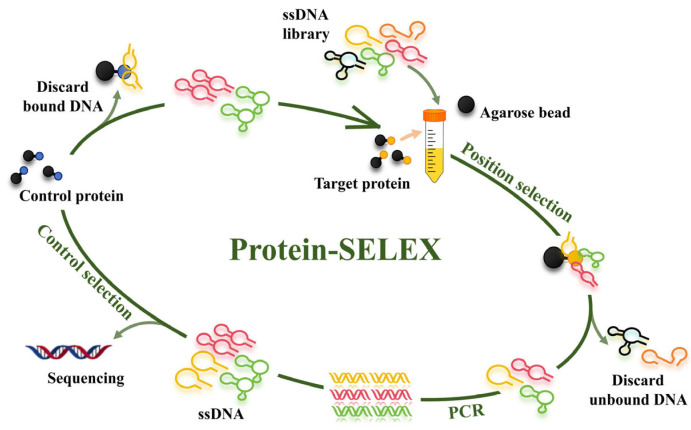
Schematic diagram of protein-SELEX screening process.

Employing SELEX technology, we meticulously screened and refined an aptamer probe, ensuring its high sensitivity and specificity for recognizing GST molecules. Our experimental strategy initially targeted S100A9, a calcium-binding protein that is prototypical of the S100 family, owing to its structural and functional parallels with GST. Through a stringent selection process, we uncovered a compelling aptamer candidate, which we designated as Seq3. This aptamer, consisting of 79 nucleotides (5’-AGCGTCGGATACCACTACTATGCCGGTCGGGGGTTGGGGATCTCTCTTGGGGGAGGGTTATCATGGAGTTCGTGGTCAG-3’), showed exceptional promise for its targeted binding to GST molecules. We observed that when Seq3 was labeled with AF488 fluorescein (Fam-Seq3), it demonstrated a strong and specific binding affinity towards GST, even in the presence of S100A9, underscoring its selective recognition capabilities. As illustrated in Figure 1, the recombinant protein and SA beads as a whole were used as targets for positive screening, while the no-load GST tag and the whole microbeads were used as controls for reverse screening. Following positive and counter selection, free ssDNA was separated from protein-bound ssDNA through centrifugation, effectively enriching the aptamer with the highest affinity for GST. Subsequently, PCR amplification was carried out until the library reached saturation, at which point it was forwarded to Sangon Biotech (Shanghai) for further processing. After undergoing eight rounds of rigorous selection, four aptamers were identified, each demonstrating a high affinity for GST recognition at the nanomolar level (Kd). These aptamers, characterized by their exceptional binding properties, underscore the successful outcome of the selection process.

**Figure 2:**
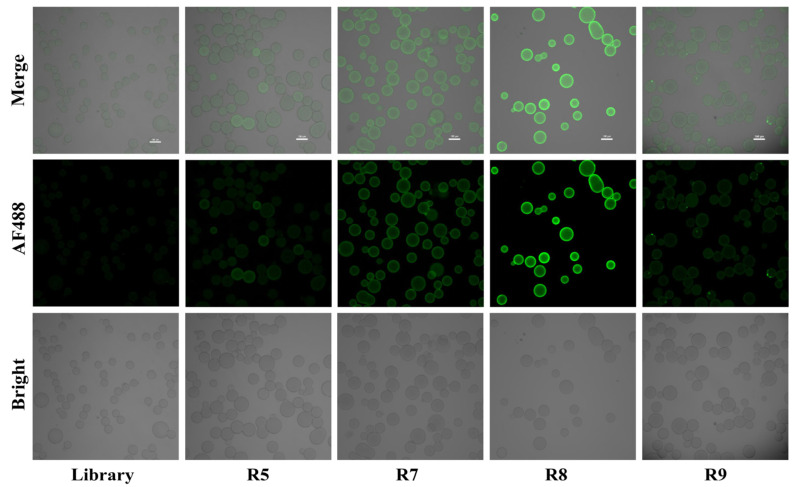
Confocal imaging fluorescence intensity verified the enrichment degree of the library.

Initially, we used recombinant protein (S100A9) as the target and the tag (GST) as the control to obtain aptamers, expecting to obtain aptamers that can specifically recognize S100A9, but in fact, the sequence obtained by screening was specifically recognized with the GST control protein (Figure 2). We were surprised to find that the results were inconsistent with the reference literature [22]. In some of their papers, Raunak Jahan et al. identified an intriguing aptamer candidate for situations similar to our study. In the SELEX experiment on PD-L1 protein, the aptamer was screened to maintain high affinity even in the absence of protein [23]. In previous studies, we used cell-SELEX technology to screen DNA aptamers of metastatic prostate tumor cells with high affinity and specificity for both target cells and negative cells [24]. Consequently, we posit that the underlying reasons for this outcome may reside in the following factors: (1) Uncertainty in the Interaction Mechanism and Specific Binding Sites: During the screening process, the modes of action between the aptamers and their targets, along with the precise sites of interaction, remain ambiguous. Inadequate elution operations and improperly calibrated screening pressure failed to effectively remove the ssDNA non-specifically bound to GST. (2) Differential Molecular Weights and Labeling Impact: In the positive screening phase, the S100A9 and GST complex were designated as the positive control. S100A9, with a smaller molecular mass (13 kDa), was dwarfed by GST, a significantly larger label protein (26 kDa). This disparity in size not only rendered GST a bulky tag for S100A9 but also increased the probability of ssDNA binding to GST due to the larger surface area available for interaction. (3) Structural Changes and Comparative Analysis with Negative Controls: Owing to structural alterations and other influences, the distinctions between the protein complex and the negative controls might have been more pronounced than those with S100A9 alone. This could have skewed the selection process, leading to the aptamer being recognized by GST rather than S100A9. These factors collectively may have resulted in the selected aptamer demonstrating specificity for GST instead of S100A9.

### 3.2. Selectivity of DNA Aptamer Candidates

Laser scanning confocal microscopy was used to detect the enrichment of fluorescence-labeled libraries of SA beads with target proteins in each round of screening products. Screening may be considered for termination under any of the following conditions: when the library enrichment capacity fails to improve for two consecutive rounds, specificity meets the experimental requirements, or the library’s enrichment capacity shows a decline compared to the previous round and further screening does not significantly enhance the binding rate. Observing that the affinity (Kd) fails to significantly improve over several consecutive rounds suggests that the screening is approaching saturation. Any of these signals can indicate that the screening should be halted, with repeat experiments serving as supplementary judgment points for consideration. After incubating the initial library and the fifth-, seventh-, eighth-, and ninth-round libraries with the target protein at 37 °C for 40 min, the fluorescence expression of FAM was shown in Figure 2. As the number of rounds increases, the fluorescence intensity on the surface of the SA beads gradually becomes stronger. In the eighth round, the library reached saturation state, the aptamer with high affinity and specificity to the target protein accounted for the largest proportion, and the fluorescence showed a downward trend in the ninth round, indicating that the aptamer in the library was lost with further selecting.

After eight rounds of selection and enrichment, the top 20 repetitions were obtained through high-throughput sequencing. The acquired sequences are listed in Table 1. As shown in Figure S3 (Supplementary Materials), aptamers can be classified into four families based on their sequence homology, with the homologous regions being highlighted. Figure S4 (Supplementary Materials) illustrates their binding affinity towards S100A9 and GST proteins. Among them, Seq3, Seq4, Seq7, and Seq20 were selected for further characterization purposes. Subsequently, Mfold software was employed to simulate and predict the secondary structures of these four aptamers, as depicted in Figure S5 (Supplementary Materials).

**Figure 3:**
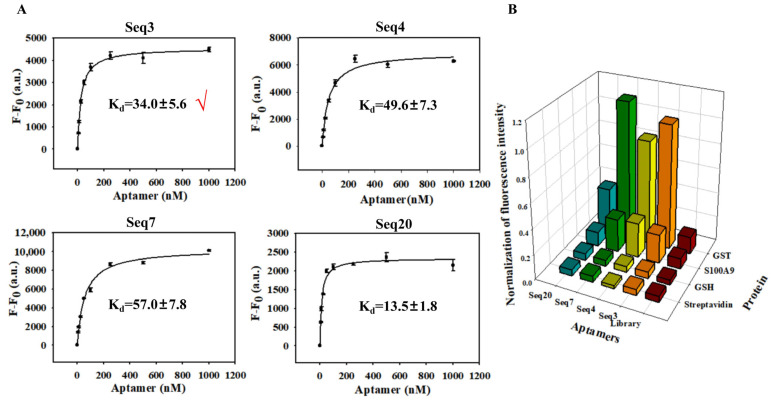
Determination and specificity of sequence dissociation equilibrium constant: (**A**) the saturation curves and Kd values of Seq3, Seq4, Seq7, and Seq20; and (**B**) investigation of sequence specificity.

**Figure 4:**
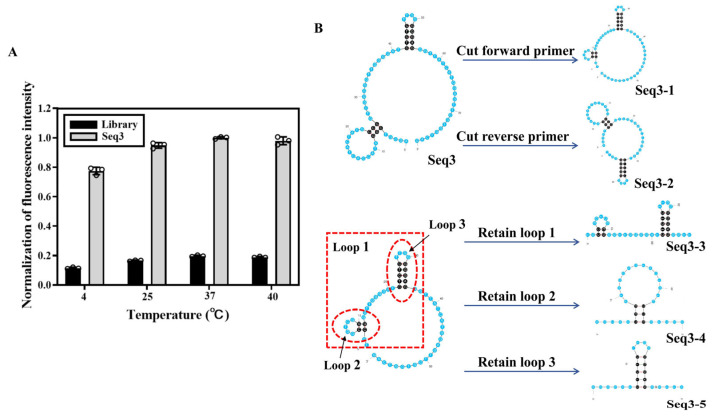
Effect of conditional optimization on the binding ability of Seq3 sequences. (**A**) The binding ability of temperature to Seq3 sequence was investigated. (**B**) Sequence optimization of aptamer Seq3, showing its secondary structure as predicted using the Mfold software (version 2.1).

The sequences were prepared into reaction systems with eight concentration gradients, and the average fluorescence intensity resulting from non-specific adsorption by SA beads was excluded. A single-point adsorption model Y = BmaxX/(Kd + X) was used to simulate the Kd of the aptamers. As illustrated in Figure 3A, the Kd of all four aptamers and GST fell within the nanomolar range, and their specific recognition capabilities were evaluated using GSH, S100A9, and Streptavidin as controls (Figure 3B). Despite Seq20 exhibiting the smallest Kd value, its confocal laser imaging combined with GST resulted in the lowest saturation fluorescence intensity. Taking all factors into consideration, Seq3 was selected for sequence optimization in subsequent experiments. Maintaining protein activity is crucial during the SELEX process. We elected incubation temperatures of 4 °C, 25 °C, 37 °C, and 40 °C to investigate the impact of temperature on sequence binding ability. The fluorescence intensity of the Seq3 sequence analyzed by ImageJ gradually increased with the increase in temperature, reaching a peak value at 37 °C and slightly decreasing at 40 °C but still maintaining a high level (Figure 4A). Therefore, it can be concluded that although temperature does have an effect on the binding ability between Seq3 and GST, these four temperatures still maintain superior recognition abilities compared to the initial library, making them suitable as molecular recognition probes.

**Table 1:** Results of the 8th round of screening library sequencing.

Aptamers	Sequences (5′–3′)	Repetition
Seq1	GGTCGGGGGTGTTCATTCTTCTTGGGGGAGGGCGGGCCGT	7920
Seq2	CACGGTGGGGGGCGGGAATTCTCTTGTTGGGGGGTGGGCT	4054
Seq3	TGCCGGTCGGGGGTTGGGGATCTCTCTTGGGGGAGGGTT	2268
Seq4	AGGGCGGGGGGGGGCTCTCTTGCTACTGGGGGAGGTTTA	1935
Seq5	CTCGCCGAGAACTCAGCTGCAGGAGTGAACCGTCCGCACG	1424
Seq6	CCACCCGCCGGTCGTGGGCCCTCTCAGTGGCTGTAGCATT	961
Seq7	TGGCGGGGGTCGTAGTCGGGGGCTACTACTGGGGGGGGGC	831
Seq8	ATTCGGGGTTGAGGGGGTATGTTATTGGGGGTGGGTGGGC	646
Seq9	GGTCGGCTAGGGGGCTGTTCAGTACGGGGGAGGGCGGGCC	579
Seq10	AGGGCGGGGGGGGGGCTCTCTTGCTACTGGGGGAGGTTTA	500
Seq11	GCGTGGATGGGTGGGGGTCACACTTGGGGGTTCGGGTGGA	483
Seq12	GCTGCAGCAAAGCGCCCGCACGACCATCTGATGGCTGCCC	415
Seq13	CCGGGACTCCGGTCTCCTCGCTGCAGCTTCGCGCCCGCAC	382
Seq14	ACTTCGGGGTCAGTCTCGGGGTCTTCTTGGGGTTGGGGTT	367
Seq15	GCTGCAGCAAAGCGCCCGCACAACCATCTGATGGCTGCCC	338
Seq16	TGTCTGGGTGGGTTTTCTATTTTCATGGGGTGGGCTTATT	335
Seq17	ATCCAGCTCGGGGCGGTGGGTTTTTGGGCGTAGGTACAGA	275
Seq18	TAGTCATTGGGGCGGGTTGCTTATTGCTTATGGGTGGGCT	251
Seq19	CCGCTGCAGCGTGCGCTGACTGGTGCACGAGCCCGCACTT	220
Seq20	CTCCTCTTCACCACAGCTCCGACGCATCACGACATGAGGG	216

### 3.3. Sequence Optimization of Aptamer Seq3

Post-SELEX optimization is a crucial step for obtaining practical aptamers with reliable affinity at a low cost, mainly by removing unwanted fragments in ssDNA sequences to improve the application value of nucleic acid aptamers [25,26]. The aptamer Seq3 is predicted to contain a large ring with two smaller stem ring structures (Figure 4B). In general, not all the nucleotides are necessary for target binding. The unnecessary ones are not beneficial for structural stability and practical usage of the aptamer [27,28,29,30]. Therefore, we minimized Seq3 by gradually removing the nucleotides from its two ends. Several strategies were applied to optimize the aptamer Seq3, including removing the fixed primer regions, removing the exterior loop nucleotides in the predicted secondary structure, or retaining the small hairpin structures; as a result, the truncated sequences Seq3-1 to Seq3-5 were obtained (Figure 4B and Table 2).

**Figure 5:**
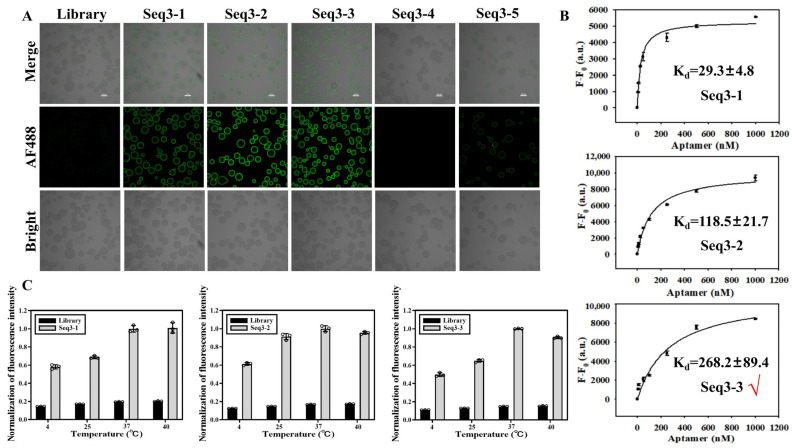
Investigation of sequence affinity after optimization. (**A**) Confocal imaging was used to investigate the sequence binding ability after optimization. Scale: 100 μm. (**B**) The modified sequence design method of Seq3-3 truncated the front and rear primers at the same time and still maintained a high fluorescence intensity level. (**C**) The effect of temperature on the combination of optimized sequences was investigated. Red mark: Seq3-3 had the best activity in combination with GST, so it was selected to continue the follow-up experiment.

Optimizing the base composition and secondary structure of aptamers is essential for ensuring their target specificity, high affinity, and selectivity towards specific molecules [31]. Through precise base composition optimization, aptamers are endowed with the exact sequence necessary for high-precision recognition and binding to specific regions of their target molecules [32]. Alterations in the secondary structure, such as the formation of hairpin and stem-loop structures, significantly enhance the aptamer’s binding affinity to the target molecule while diminishing non-specific binding, thereby improving overall target specificity and efficiency [33]. The superior performance of Seq3-1, Seq3-2, and Seq3-3 in target protein identification, juxtaposed with Seq3-5’s reduced, yet still notable, potential as a target (Figure 5A), highlighted a common motif among the target proteins: a compact stem-loop structure. This observation underscores the motif’s critical role in the aptamer–GST interaction, leading us to prudently choose the trio of Seq3-1, Seq3-2, and Seq3-3, which combined the most promising outcomes, for follow-up investigations. In Figure 5B, confocal fluorescence imaging was employed to determine the dissociation equilibrium constants of reaction systems incubated at 37 °C with different concentrations of Seq3-1, Seq3-2, and Seq3-3, along with their respective target proteins. The obtained Kd values were consistently within the nanomolar range, indicating high affinity between these sequences and their targets. In addition, the optimal incubation temperature that yielded the best binding capacity of GST was found to be 37 °C (Figure 5C). Therefore, these optimized sequences exhibit excellent temperature tolerance and can serve as effective molecular recognition probes in biomedical applications, expanding the utility of aptamers. Among them, Seq3-3 (TGCCGGTCGGGGGTTGGGGATCTCTCTTGGGGGAGGGTT, 39 bases) showed the best performance and bound GST with well-binding activity.

### 3.4. Double-Strand Fluorescent Aptamer Probe for Detection of GST

**Figure 6:**
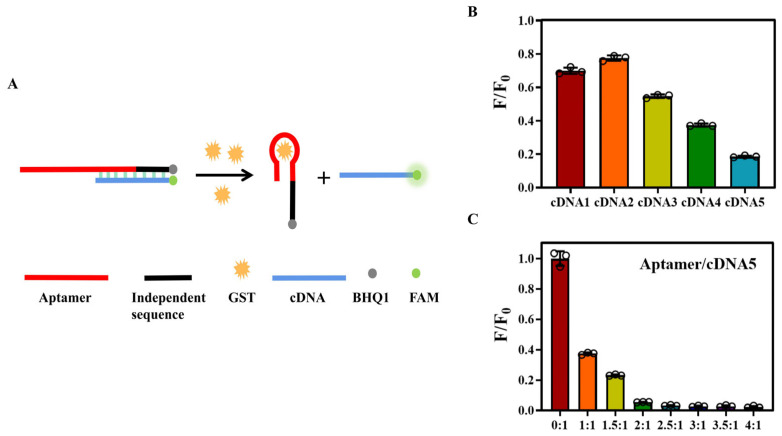
Design of double-strand fluorescent aptamer probe: (**A**) schematic diagram; (**B**) screening of cDNA; and (**C**) aptamer/cDNA5 ratio optimization.

Detection methods based on aptamers include colorimetric assays [34,35,36], the mixed “sandwich” method [37], the electrochemical method, and the fluorescence method [38,39]. Among these techniques, fluorescence-based assays [40,41] are widely utilized due to their simplicity, speediness, and lack of washing steps. When the aptamer probe interacts with the target, it undergoes a conformational change, resulting in a modulation of the fluorescence signal, which serves as the output signal. Therefore, we devised a double-stranded fluorescence probe for detecting GST (Figure 6A). The ingenious design involves supplementing the aptamer Seq3-3 sequence with antisense oligonucleotides by extending an unrelated sequence at its 3’ end (aptamer labeled BHQ1 and cDNA labeled FAM), thereby preventing blockage of the probe’s active site and impeding interaction with the target. Sensors with similar principles have been widely reported [42,43,44,45,46,47], but the sensor we designed has great advantages in performance comparison and practical application potential. In the presence of GST, competition between the target protein and cDNA for the aptamer leads to dissociation of the double-stranded structure, transforming the aptamer/DNA complex into an aptamer/target complex and enabling recovery of fluorescence signal. This method offers flexibility in design, simplicity in synthesis, convenience, and speediness, making it a promising general approach for target detection.

The key determinant for protein detection using a double-stranded fluorescent aptamer probe lies in the ability of the target protein to displace the aptamer from its structure. Therefore, it is crucial to have an appropriate cDNA length. We designed five cDNAs with varying lengths: 11, 12, 13, 14, and 15 bases, respectively. The fluorescence value (F0) of each cDNA was determined using steady-state near-infrared fluorescence before complementing with aptamers (F). A larger F/F0 value indicates lower quenching efficiency and vice versa. As shown in Figure 6B, increasing the number of bases within our design range improves quenching effectiveness; hence, we selected cDNA5 for probe assembly. Additionally, maintaining an optimal concentration ratio between double chains is vital for accurate protein detection. During the optimization experiments on aptamer/cDNA5 ratios, we found that increasing concentration ratios were positively correlated with improved quenching efficiency until reaching a plateau at 2:1 (Figure 6C).

**Figure 7:**
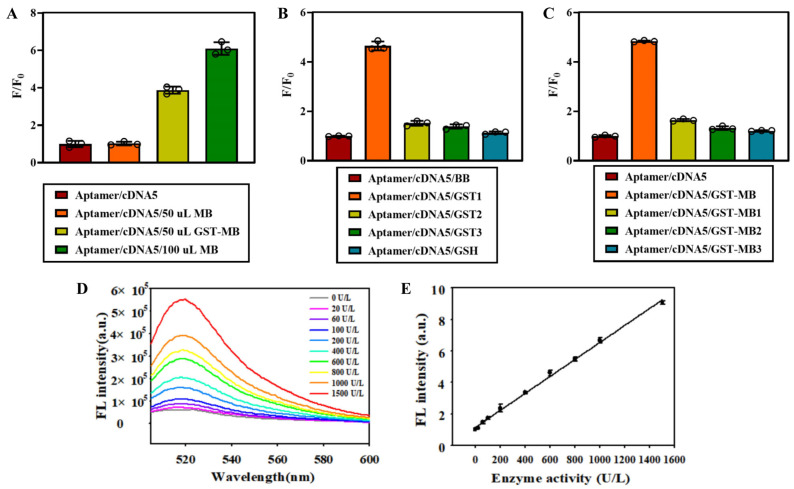
Study on the activity of double-strand fluorescent aptamer probe for GST: (**A**) detection of GST on agarose microbeads; (**B**) detection of GST in eluents; (**C**) detection of GST on elution remaining microbeads; (**D**) fluorescence spectrum of GST quantitative detection; and (**E**) the standard curve in D.

The double-strand fluorescent aptamer probe, assembled under the optimized conditions mentioned above, was employed for GST detection. The fluorescence intensity of the aptamer/cDNA complex was denoted as F_0_, while that of the target GST was denoted as F. A higher F/F_0_ ratio indicates a more effective detection. Initially, we examined the presence of the GST protein immobilized on agarose microbeads (GST-MB). As depicted in Figure 7A, upon the addition of GST-MB, the fluorescence signal recovered and became stronger with increasing amounts of GST-MB. The background ratios were fourfold and sixfold for 50 μL and 100 μL, respectively. Subsequently, a binding buffer (BB) and elution buffer (EB) were used as controls to extract the GST protein solution from MBs, followed by probing three consecutive elution supernatants (Figure 7B). With each subsequent elution cycle, the fluorescence signal weakened, indicating a decrease in the concentration of the GST protein in the supernatant. This trend is further confirmed by Figure 7C, where it can be observed that the fluorescence signal is strong when no elution is performed on GST-MBs but weakens with each additional elution cycle. After one round of elution, the fluorescence value reduced significantly, suggesting high efficiency can be achieved after a single elution.

**Figure 8:**
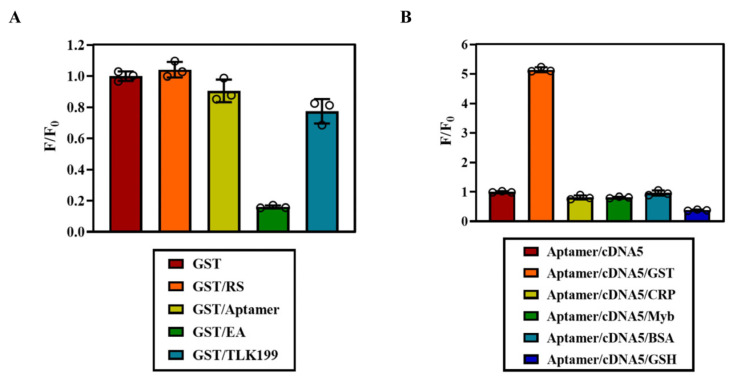
Study on the specificity of double-strand fluorescent aptamer probe for GST: (**A**) effect of aptamer on enzymatic reaction of GST; and (**B**) investigation of specificity of double-strand fluorescent probe.

Enzyme activity plays a crucial role in protein detection. If the GST protein undergoes denaturation, it not only loses its ability to participate in enzymatic reactions but also undergoes changes in its morphological structure, making it unidentifiable and unable to be targeted by the screened aptamer. Therefore, we employed a probe to quantitatively assess the activity of the GST protein. GST was diluted into corresponding activity gradients, where abscess (X) represented enzyme activity and ordinate (Y) indicated fluorescence intensity recovered by the probe. As depicted in Figure 7D,E, the range of the GST protein activity spans from 0 to 1500 U/L, exhibiting a strong linear relationship with fluorescence signal (Y = 324.6X + 68528.4, R^2^ = 0.9977), and the detection limit is 1.4 U/L. With the advantages of cheap nucleotide, simple design, and convenient synthesis, the probe is expected to be prepared into biological sample products, which can solve the shortcomings of traditional detection methods, such as high cost, strict conditions, and equipment limitations. In terms of detection medicine, the limit of the GST control group is generally 2–5U/L, and the positive group is generally higher than 10U/L. Our probe can distinguish well in sample classification and standard determination and fully meet the basic requirements of sensitive sample examination in scientific research. When testing for GST activity using an assay kit specifically designed for this purpose, there was a slight decrease after random chain or aptamer incubation reaction with minimal impact observed. However, upon the addition of active inhibitor EA, there was a significant reduction in GST activity, while no change occurred after adding active inhibitor TLK199 (Figure 8A). This discrepancy arises because TLK199 acts as a glutathione analogue prodrug present in ethyl ester form, which hydrolyzes via esterase within in vivo conditions to release active drugs inhibiting GST functions. During our in vitro experiment, TLK199 remained unhydrolyzed, resulting in no significant decrease in GST activity due to the lack of drug release necessary for inhibition purposes. Our designed probe exhibits no reactivity towards C-reactive protein (CRP), myofibrillar protein (Myb), bovine serum albumin (BSA), and GSH. It demonstrates excellent specificity towards GST while having a negligible impact on the activity of the target protein (Figure 8B).

## 4. Conclusions

In summation, glutathione S-transferase (GST)-specific aptamers were meticulously selected from a randomized single-stranded DNA library of 79 nucleotides, utilizing target protein-immobilized streptavidin agarose beads SELEX technology. Through a rigorous eight-round screening process, incorporating both forward and reverse screening, four aptamers emerged as capable of specifically recognizing GST. Prominently, the Seq3 aptamer showcased a potent specific binding ability, with a Kd value of 34 nM, demonstrating its robust affinity under conditions of 37 °C incubation. Further optimization of Seq3 involved refining its base composition and secondary structure. Through predictive modeling and experimental validation, both forward and reverse primers were pruned, culminating in a potent ligand, Seq3-3, of 39 nucleotides in length. This method of selection and truncation not only yields efficient, cost-effective, high-affinity aptamers but also presents a versatile strategy applicable to a wide range of molecular targets.

To assess the practical utility of truncated aptamer sequences, a double-stranded fluorescent aptamer probe was engineered for the in vitro detection of GST. This probe demonstrated high sensitivity within a linear detection range from 0 to 1500 U/L, achieving an impressive detection limit as low as 1.4 U/L. The sensor’s straightforward design facilitates the rapid detection of diverse target proteins by simply swapping the aptamer component. Notably, the probe’s high sensitivity and favorable fluorescence response in real-world samples hint at its application potential in protein detection, food safety, environmental monitoring, and medical diagnostics. Conclusively, our novel double-stranded fluorescent aptamer probe for GST detection represents a significant leap over existing methods, excelling in specificity, sensitivity, and versatility. Ongoing research will further refine its capabilities and expand its application across various scientific and clinical domains.

## Figures and Tables

**Table 2:** Sequences and dissociation constant (Kd) values of the truncated aptamers.

Aptamers	Sequences (5′–3′)	Repetition
Seq3	AGCGTCGGAT ACCACTACTA TGCCGGTCGG GGGTTGGGGA TCTCTCTTGG GGGAGGGTT ATCATGGAGT TCGTGGTCAG	34.0 ± 5.6
Seq3-1	TGCCGGTCGG GGGTTGGGGA TCTCTCTTGG GGGAGGGTT ATCATGGAGT TCGTGGTCAG	29.3 ± 4.8
Seq3-2	AGCGTCGGAT ACCACTACTA TGCCGGTCGG GGGTTGGGGA TCTCTCTTGG GGGAGGGTT	118.5 ± 21.7
Seq3-3	TGCCGGTCGG GGGTTGGGGA TCTCTCTTGG GGGAGGGTT	268.2 ± 89.4

## Data Availability

Data are contained within the article.

## Supplementary Materials

The following supporting information can be downloaded at: https://www.mdpi.com/article/10.3390/bios14100476/s1, Figure S1: Gel electrophoresis images of PCR annealing temperature; Figure S2: Optimization of the number of PCR cycles and validation of batch-amplified products; Figure S3: Homology analysis of candidate sequences using Clustal X; Figure S4: Binding of candidate sequences to S100A9/GST protein; Figure S5: The secondary structures of four selected aptamers.
