# Supplementary material for: A Double-Stranded Aptamer for Highly Sensitive Fluorescent Detection of Glutathione S-Transferases
Source: Biosensors (Basel). 2024 Oct 3;14(10):476. doi: 10.3390/bios14100476 (PMC11505714; doi:10.3390/bios14100476)
Supplement: Supplementary file 1 [file biosensors-14-00476-s001.zip › Supporting Information-cuiwei.pdf]

Supporting information

A double stranded Aptamer for high-sensitive fluorescent detection of Glutathione S-transferases

Wei Cui[1, †], Suping Li[1, †], Jiahao Zeng[1], Chen Li[1], Zhaofeng Li[2], Xiaohong Wen[1], Suxiao Bao[1], Yang Mei[2, *], Xiangxian Meng[3, *] and Qiuping Guo[1, *]

[1] *College of Biology, State Key Laboratory of Chemo/Biosensing and Chemometrics, College of Chemistry and Chemical Engineering, Hunan University, Key Laboratory for Bio-Nanotechnology and Molecule Engineering of Hunan Province, Changsha 410082, China*

[2] *School of Biomedical Sciences, Hunan University, Changsha 410082, China*

[3] *College of Biology, Hunan University, Changsha 410082, China*

† *These authors contributed equally to this work.*

* *Correspondence: guoqping@hnu.edu.cn. Tel.: 86-731-88821566; Fax: 86-731-88821566. Correspondence may also be addressed to Yang Mei and Xiangxian Meng. E-mail: yanmei@hnu.edu.cn, xxmeng@hnu.edu.cn.*

Contents

Figure S1. Gel electrophoresis images of PCR annealing temperature.

Figure S2. Optimization of the number of PCR cycles and validation of batch amplified products.

Figure S3. Homology analysis of candidate sequences using Clustal X. Sequence.

Figure S4. Binding of candidate sequences to S100A9/GST protein.

Figure S5. The secondary structures of four selected aptamers.

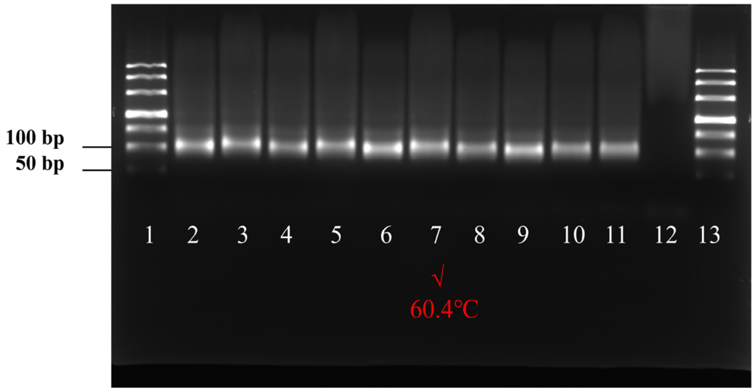

Figure S1. Gel electrophoresis images of PCR annealing temperature. 1-13: DNA Marker, 55.2℃, 55.8℃, 56.7℃, 57.8℃, 59.1℃, 60.4℃, 61.7℃, 62.9℃, 63.9℃, 64.6℃, blank, DNA Marker.

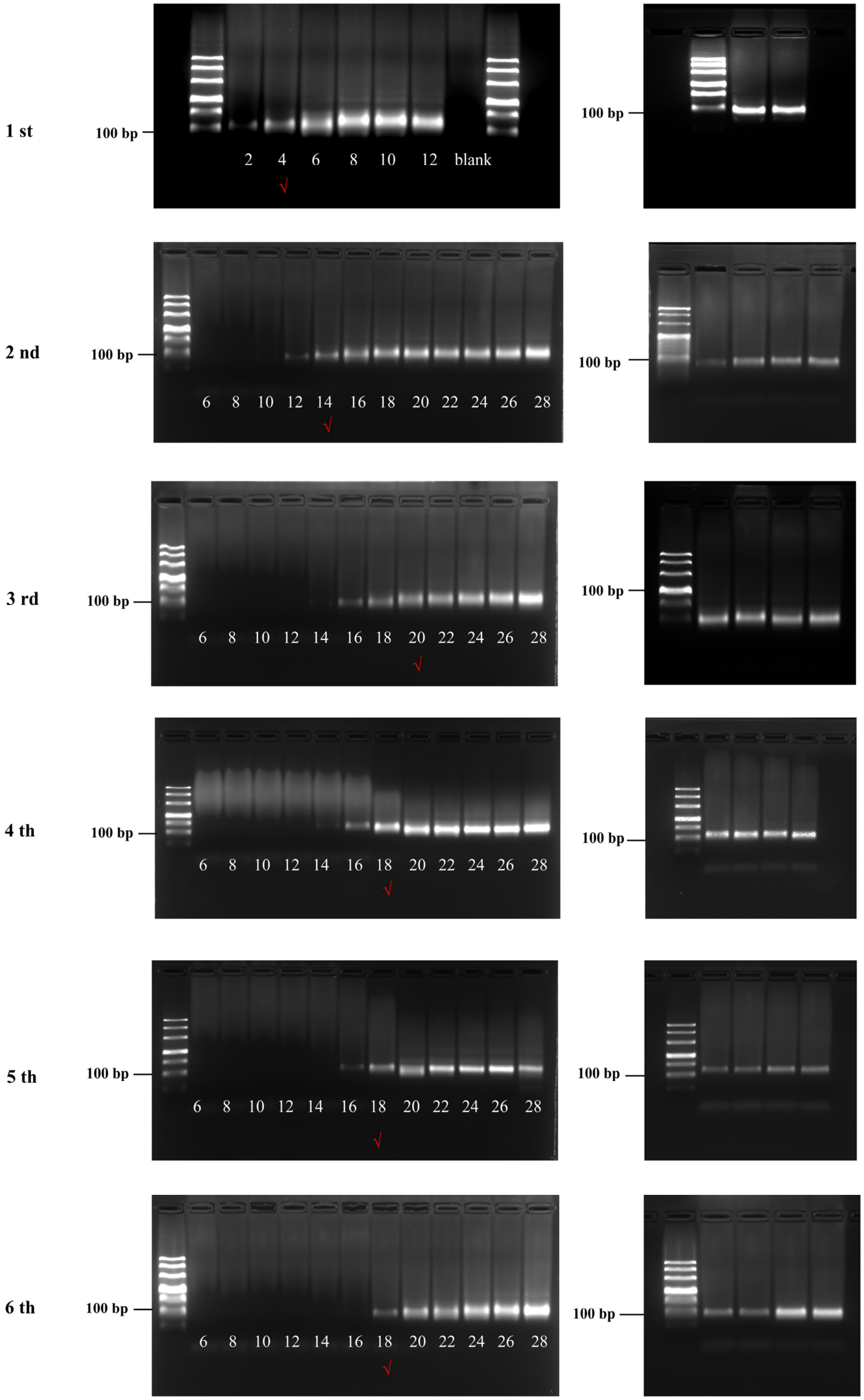
1 st
100 bp
2 4 6 8 10 12 blank
100 bp
2 nd
100 bp
6 8 10 12 14 16 18 20 22 24 26 28
100 bp
3 rd
100 bp
6 8 10 12 14 16 18 20 22 24 26 28
100 bp
4 th
100 bp
6 8 10 12 14 16 18 20 22 24 26 28
100 bp
5 th
100 bp
6 8 10 12 14 16 18 20 22 24 26 28
100 bp
6 th
100 bp
6 8 10 12 14 16 18 20 22 24 26 28
100 bp

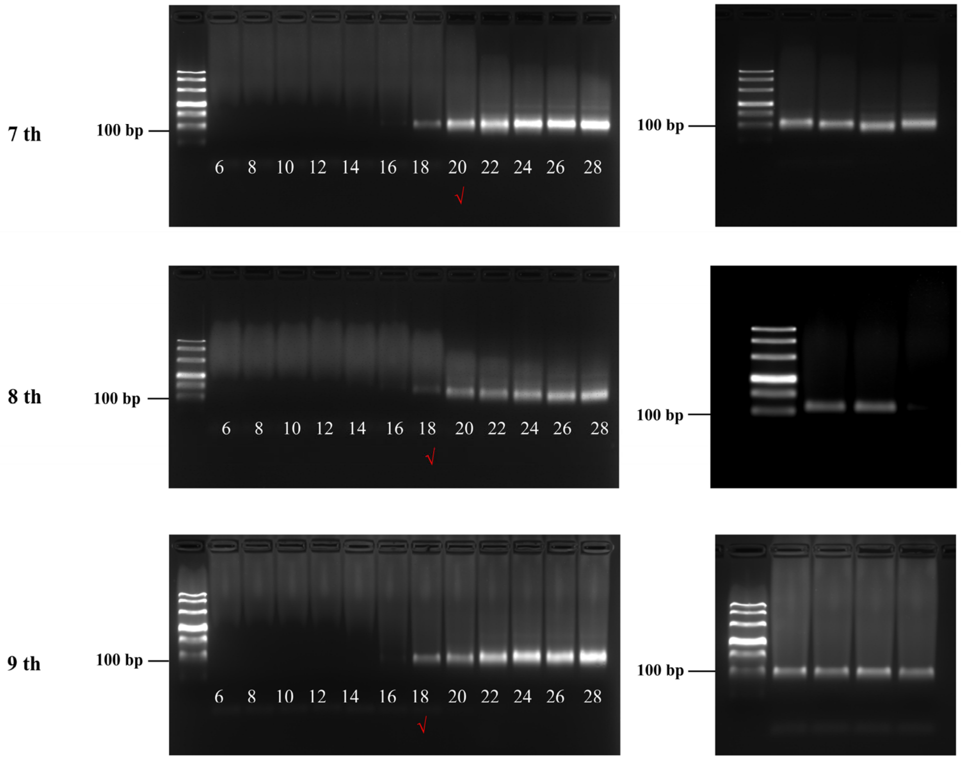

Figure S2. Optimization of the number of PCR cycles and validation of batch amplified products. On the left is the optimization of the number of PCR cycles, and on the right is the product verification results of batch amplification under the optimal number of cycles.

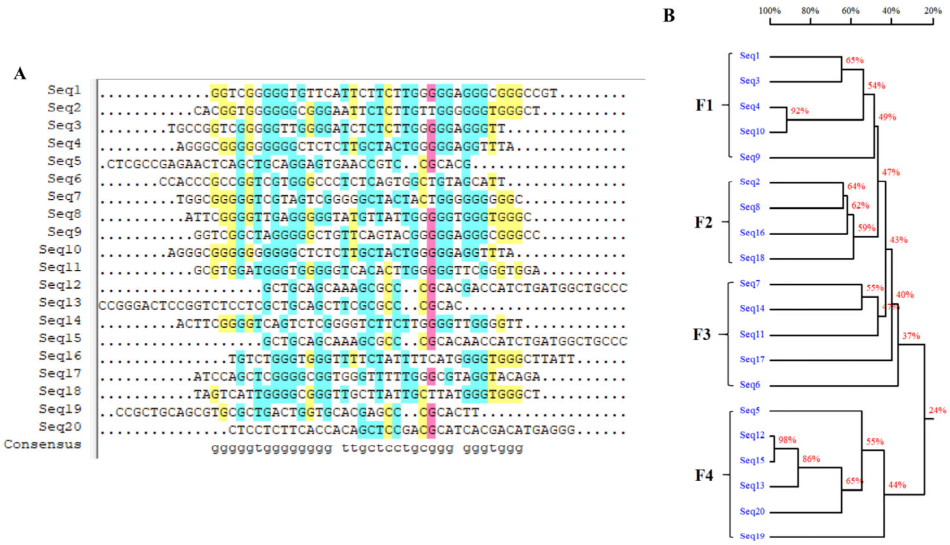

Figure S3. Homology analysis of candidate sequences using Clustal X. Sequence. Pink represents homology greater than 75%, green represents homology greater than 50%, and yellow represents homology greater than 33%. (The representative aptamers exhibit a significant presence of conservative motifs "GGG" and "TGG", which may play a crucial role in target recognition and binding.)

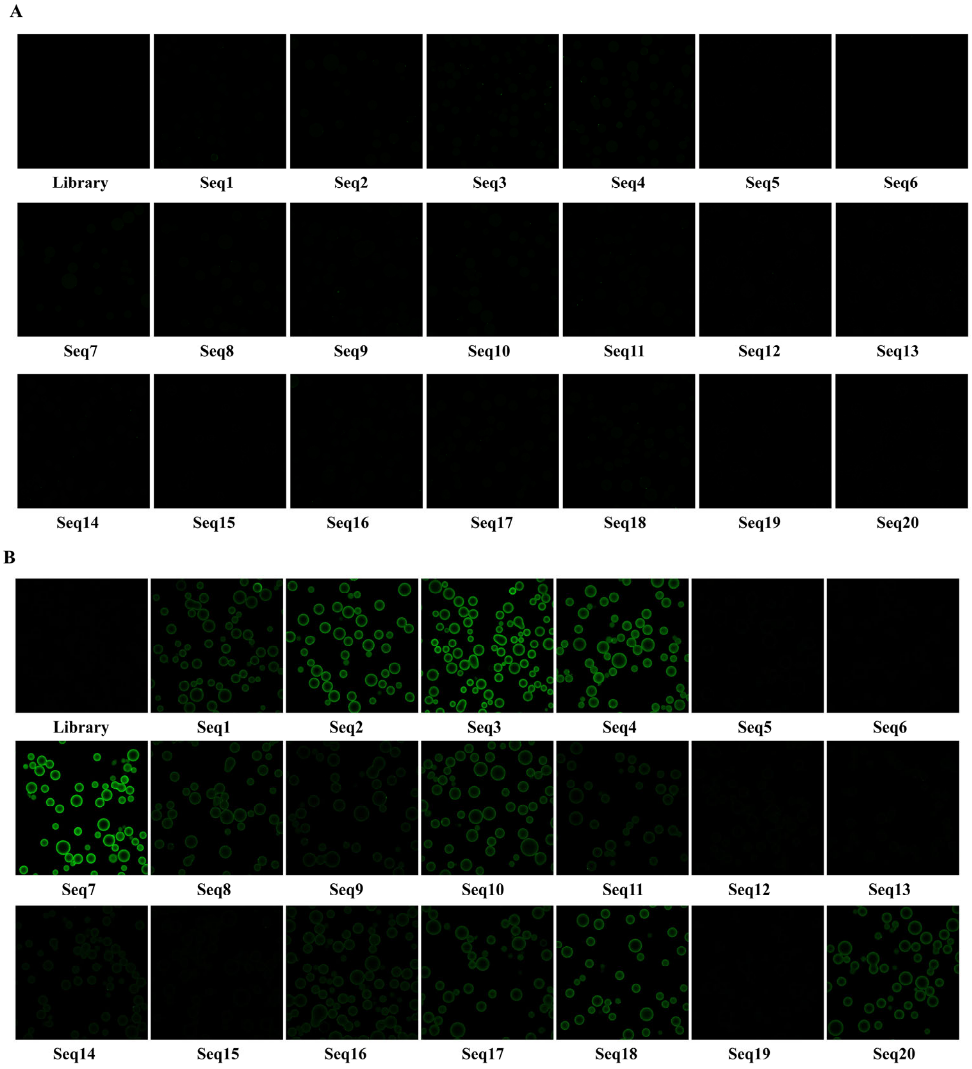

Figure S4. (A) Binding of candidate sequences to S100A9 protein. (B) Binding of candidate sequences to GST proteins.

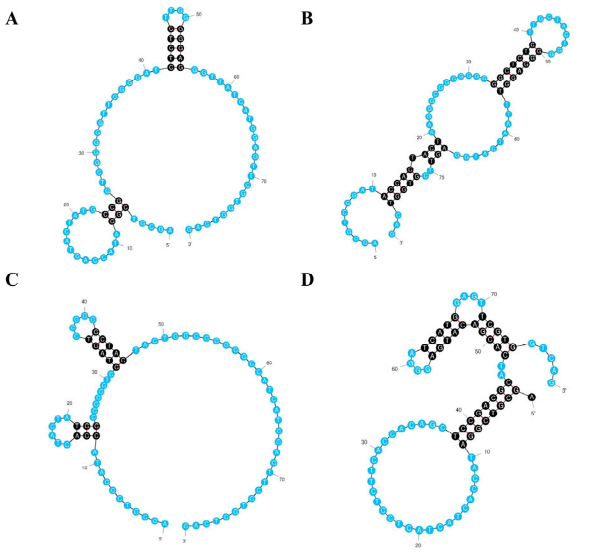

Figure S5. The secondary structures of four selected aptamers. (A) Seq3; (B) Seq4; (C) Seq7; (D) Seq20.
