# Supplementary figures and images for: A Double-Stranded Aptamer for Highly Sensitive Fluorescent Detection of Glutathione S-Transferases
Source: Biosensors (Basel). 2024 Oct 3;14(10):476. doi: 10.3390/bios14100476 (PMC11505714; doi:10.3390/bios14100476)

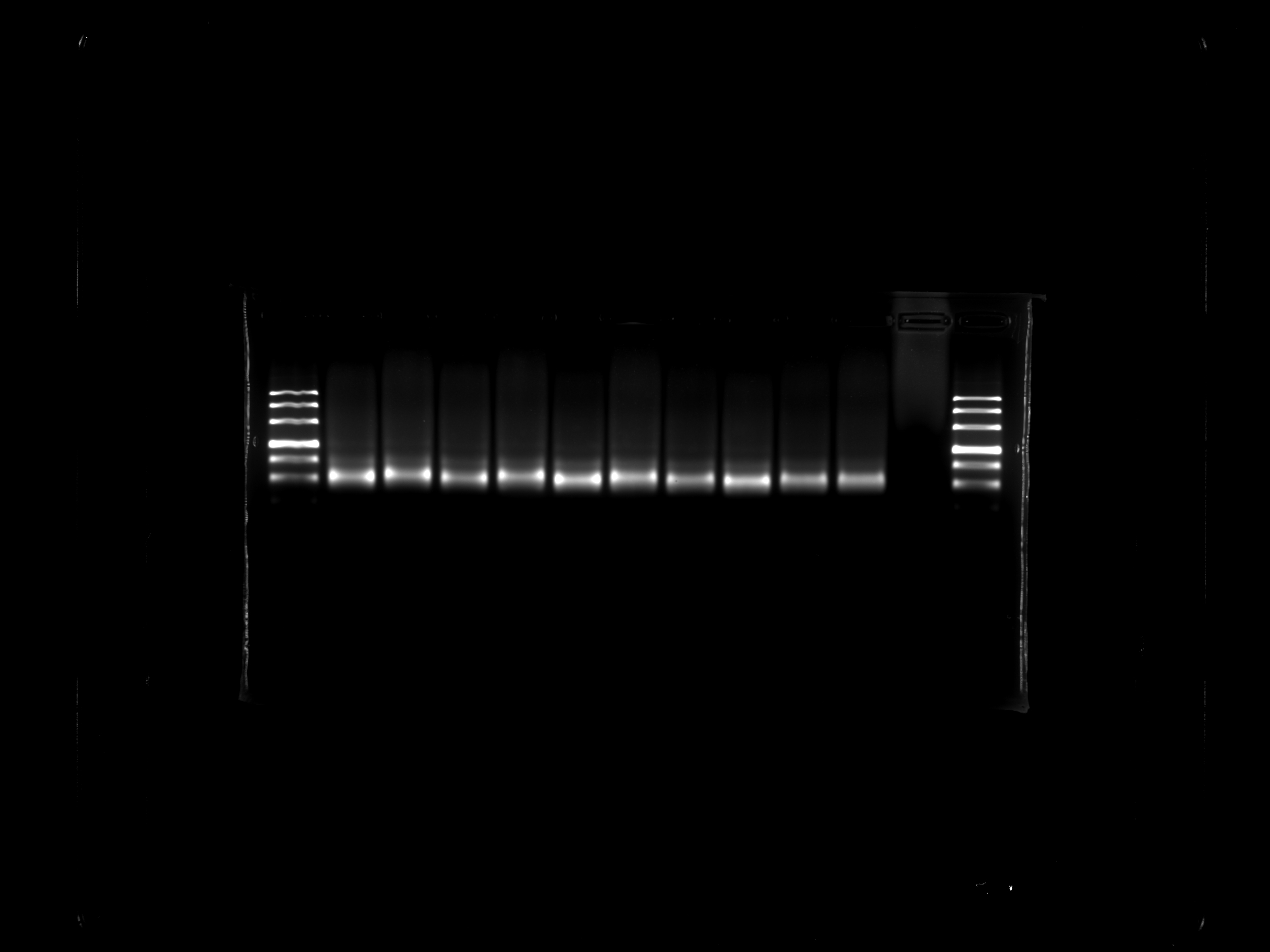

Supplement: Supplementary file 1 [file biosensors-14-00476-s001.zip › Figure S1.jpg]

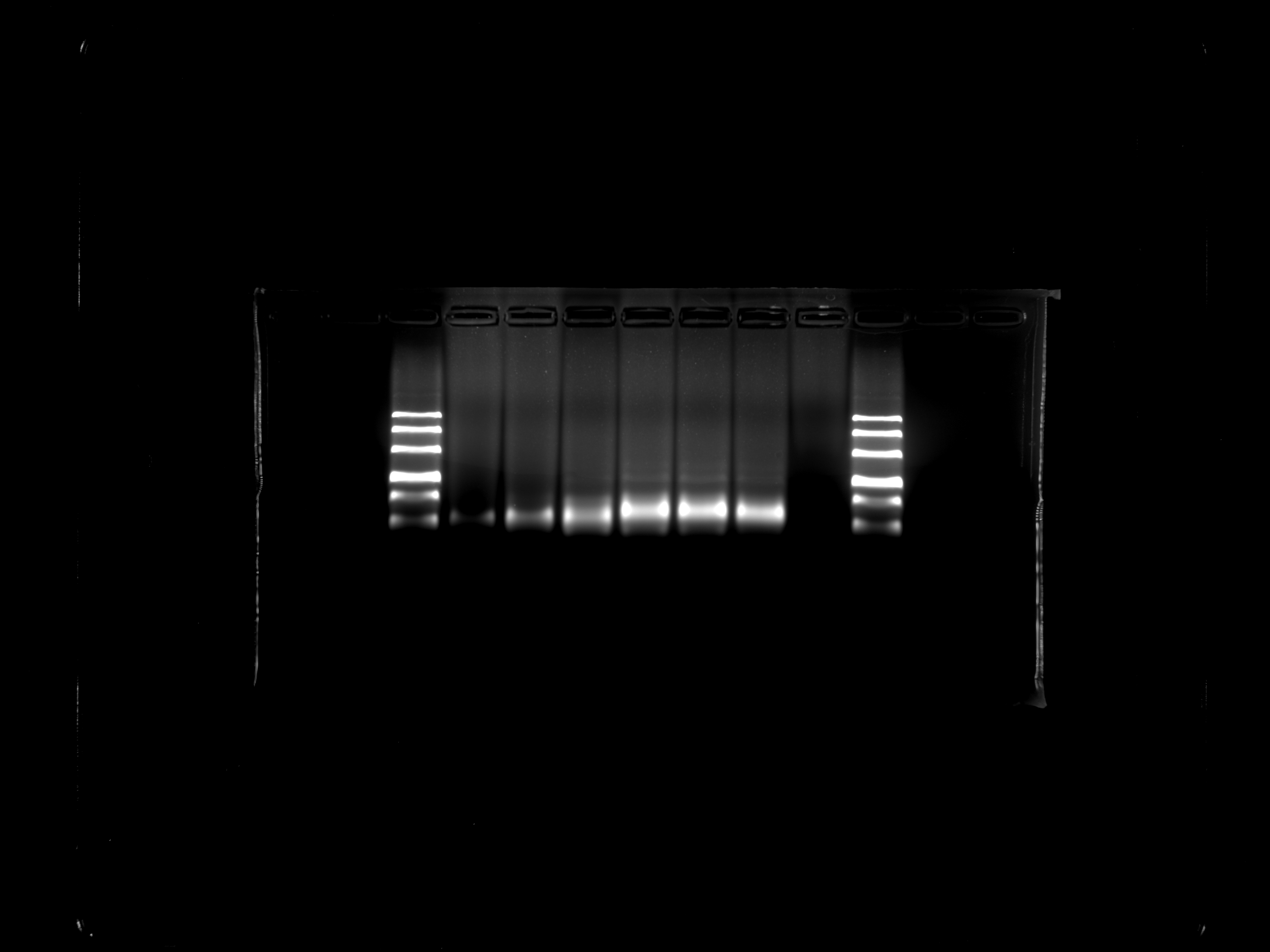

Supplement: Supplementary file 1 [file biosensors-14-00476-s001.zip › Figure S2/1 st (1).jpg]

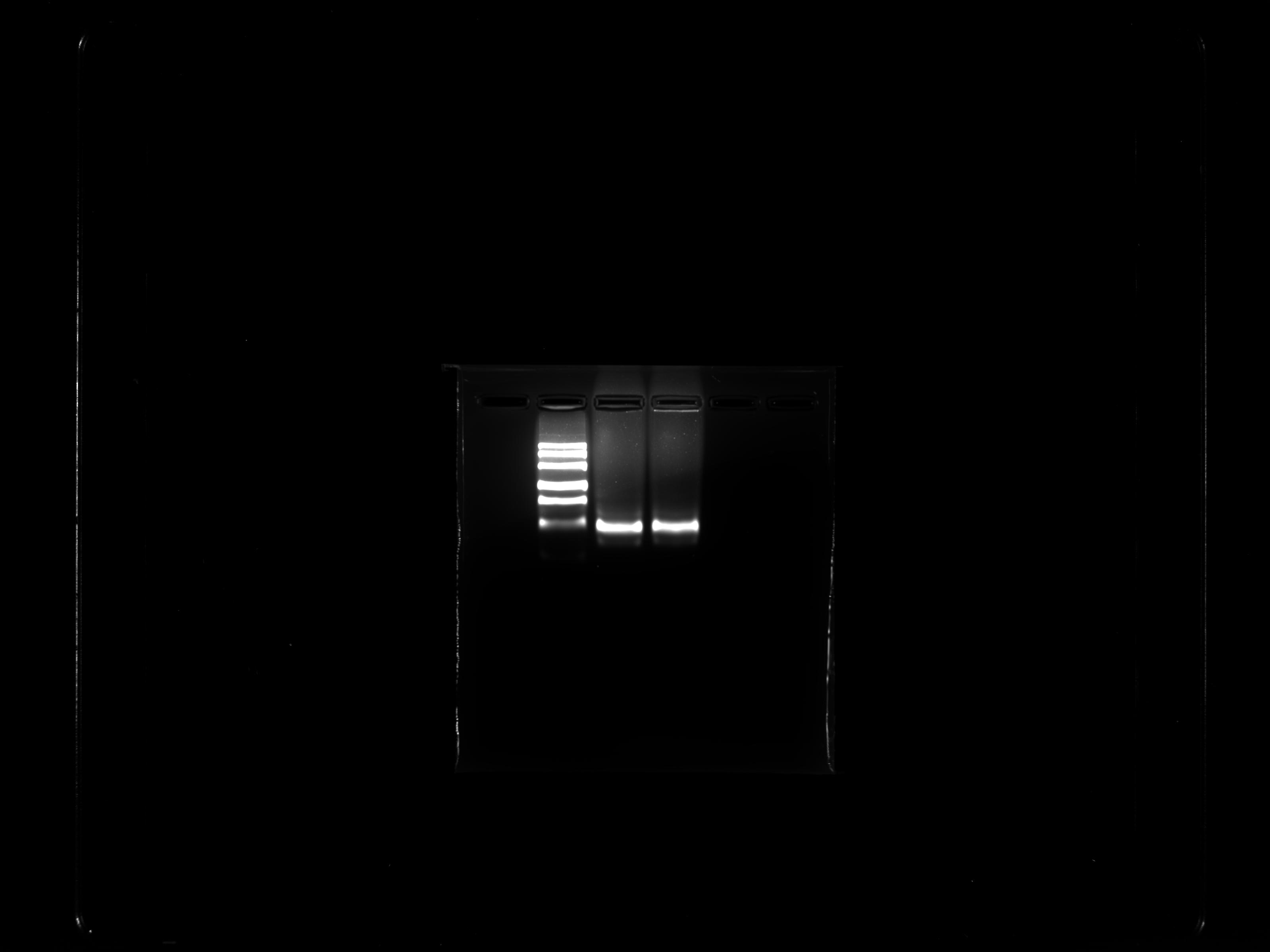

Supplement: Supplementary file 1 [file biosensors-14-00476-s001.zip › Figure S2/1 st (2).jpg]

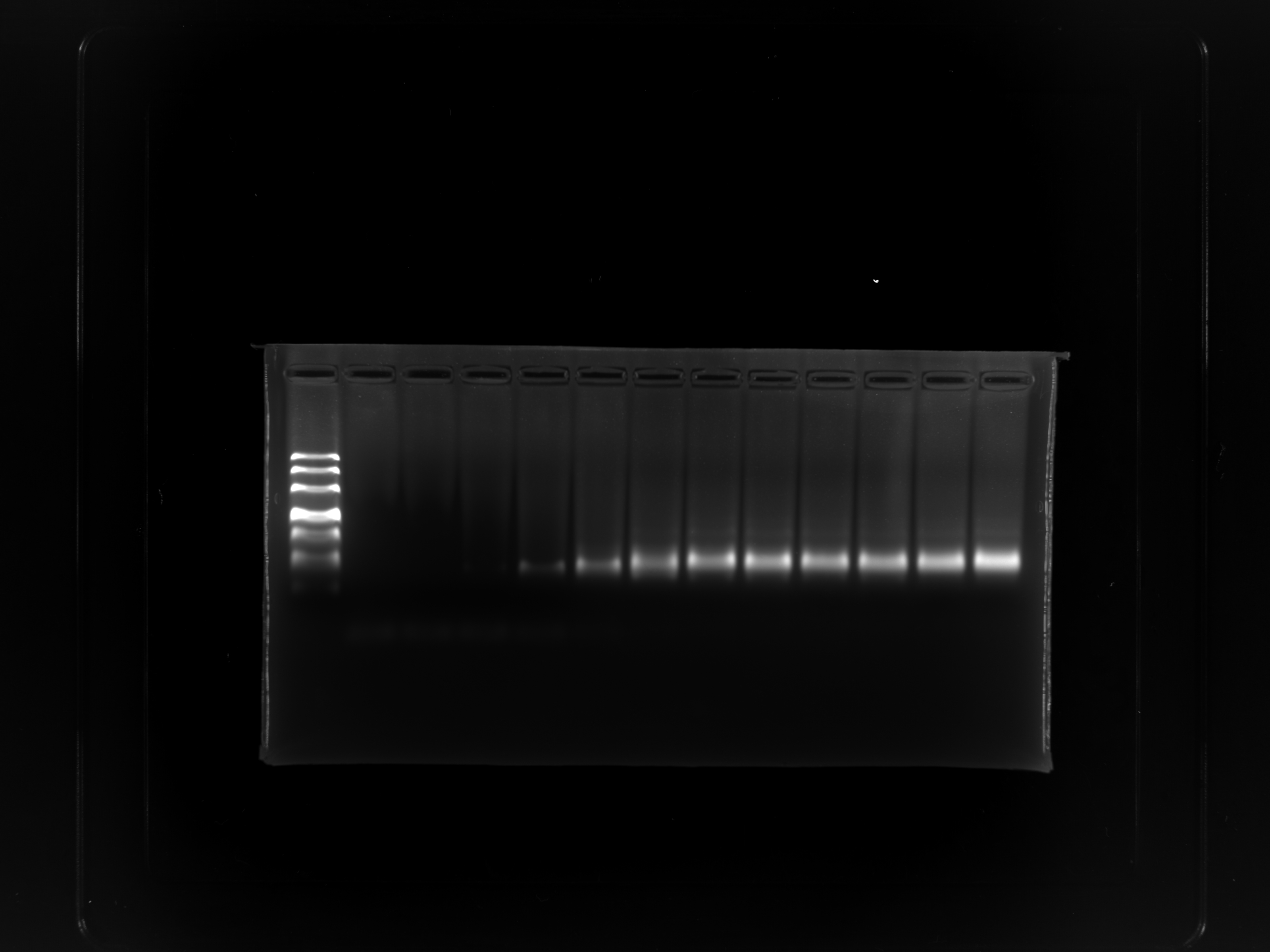

Supplement: Supplementary file 1 [file biosensors-14-00476-s001.zip › Figure S2/2 nd (1).jpg]

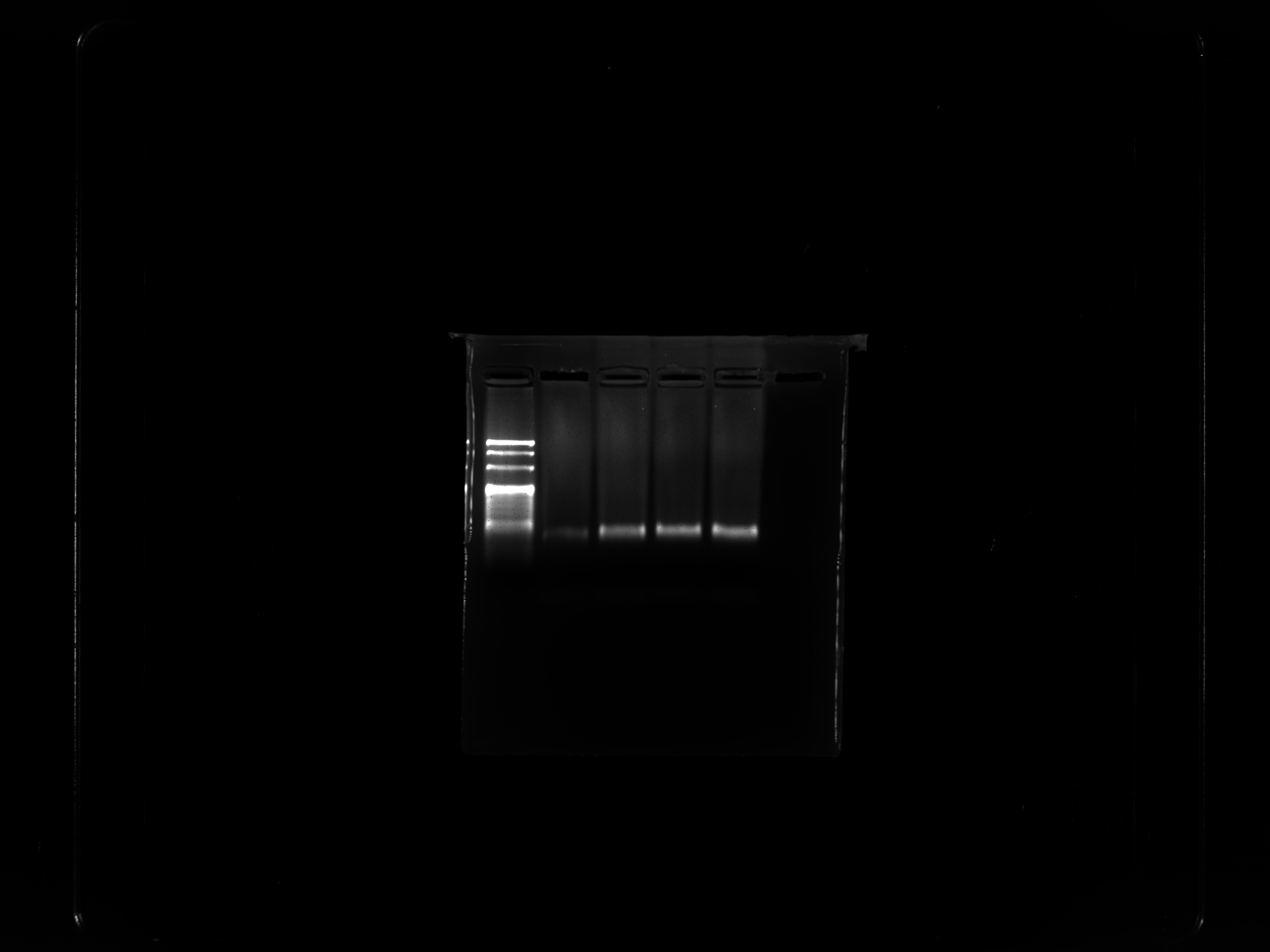

Supplement: Supplementary file 1 [file biosensors-14-00476-s001.zip › Figure S2/2 nd (2).jpg]

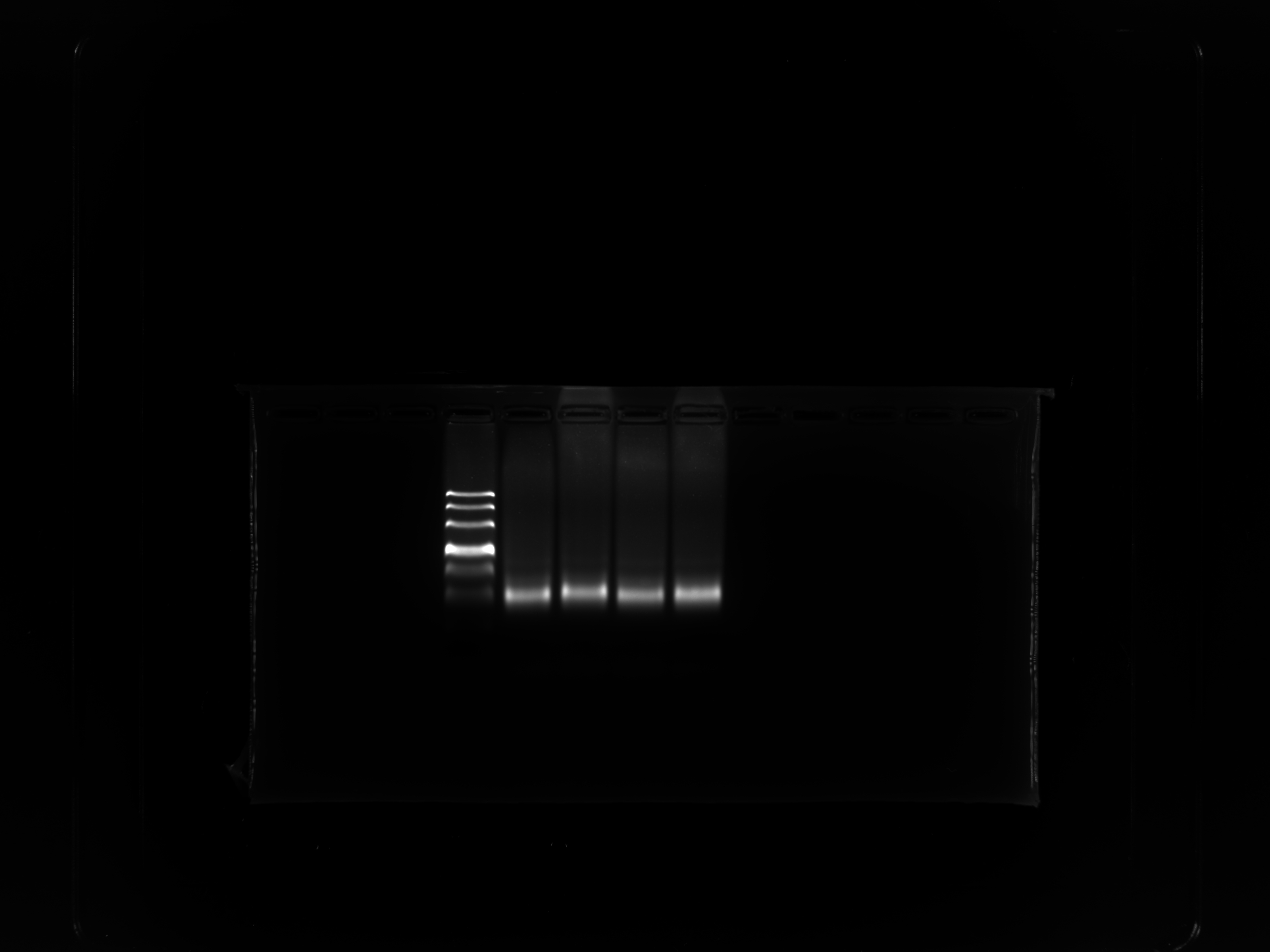

Supplement: Supplementary file 1 [file biosensors-14-00476-s001.zip › Figure S2/3 rd (1).jpg]

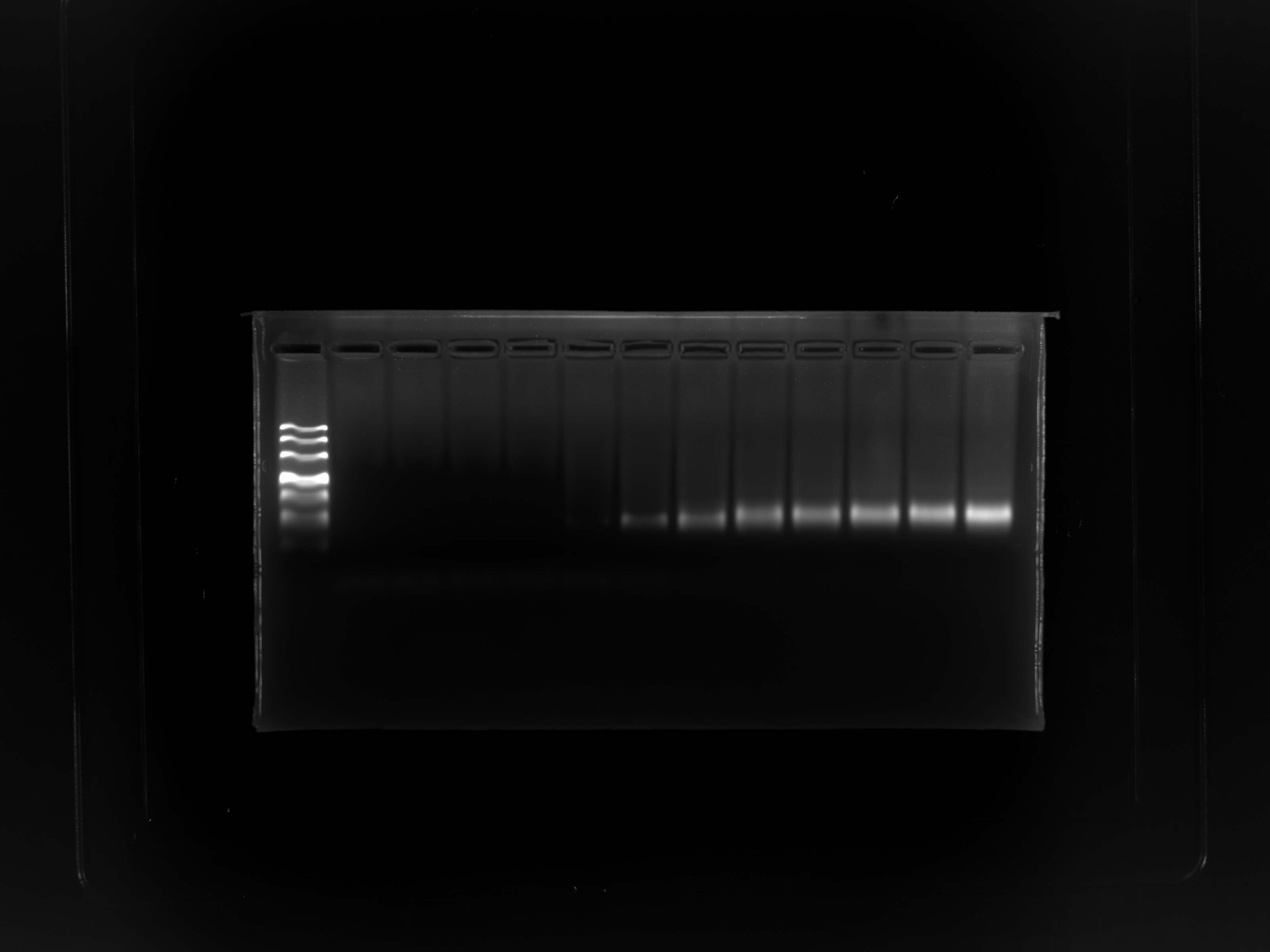

Supplement: Supplementary file 1 [file biosensors-14-00476-s001.zip › Figure S2/3 rd (2).jpg]

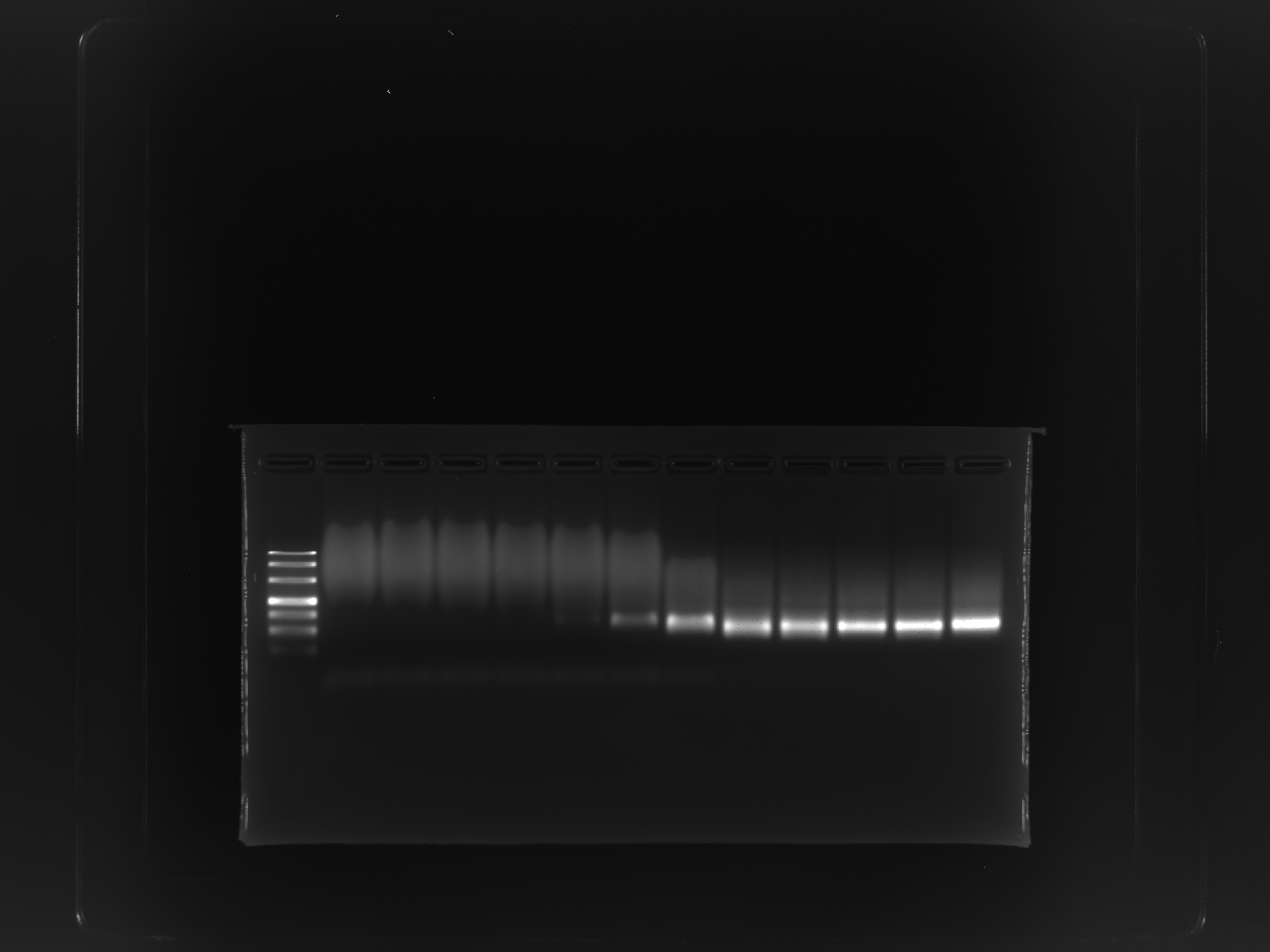

Supplement: Supplementary file 1 [file biosensors-14-00476-s001.zip › Figure S2/4 th (1).tif]

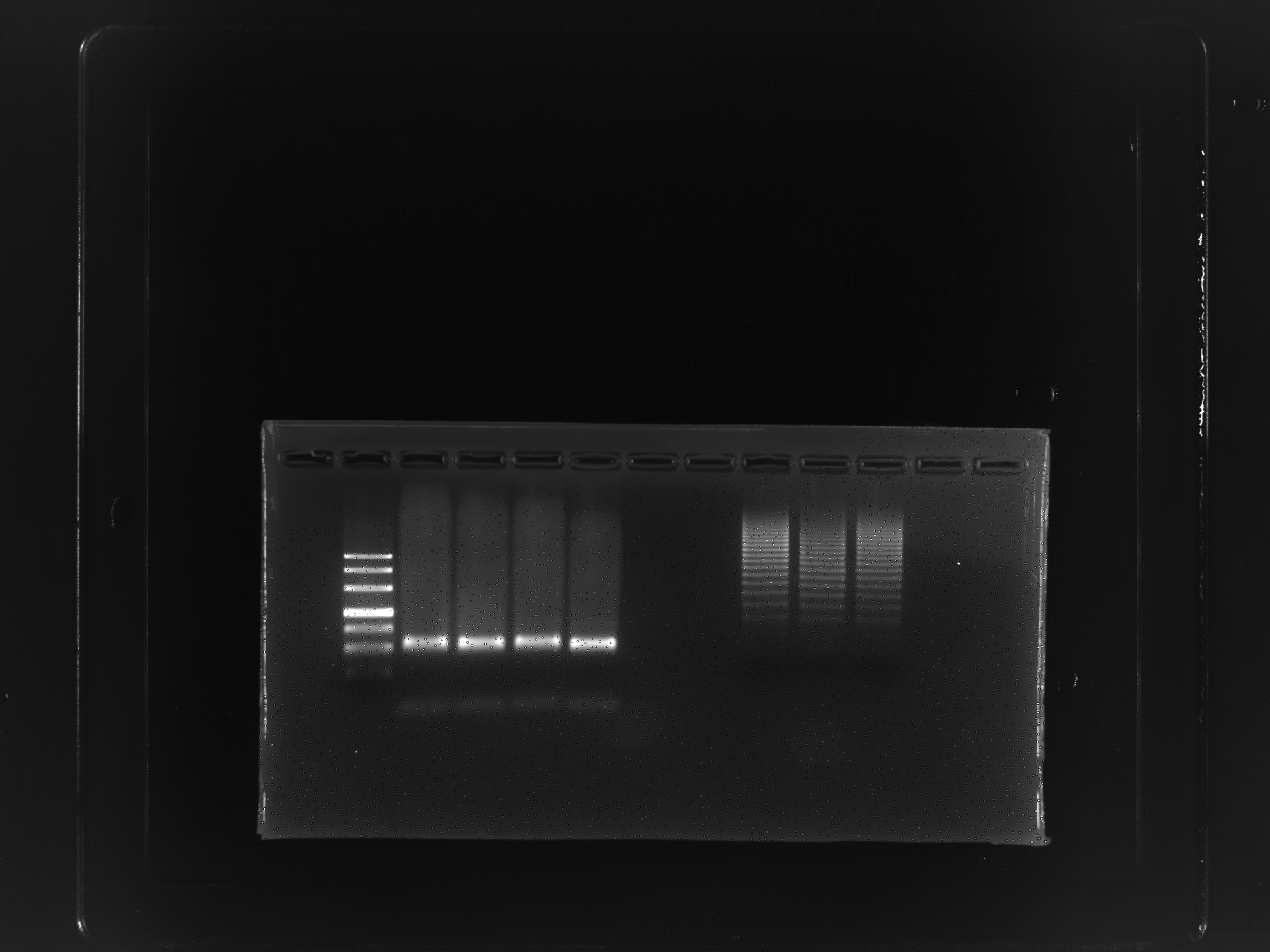

Supplement: Supplementary file 1 [file biosensors-14-00476-s001.zip › Figure S2/4 th (2).tif]

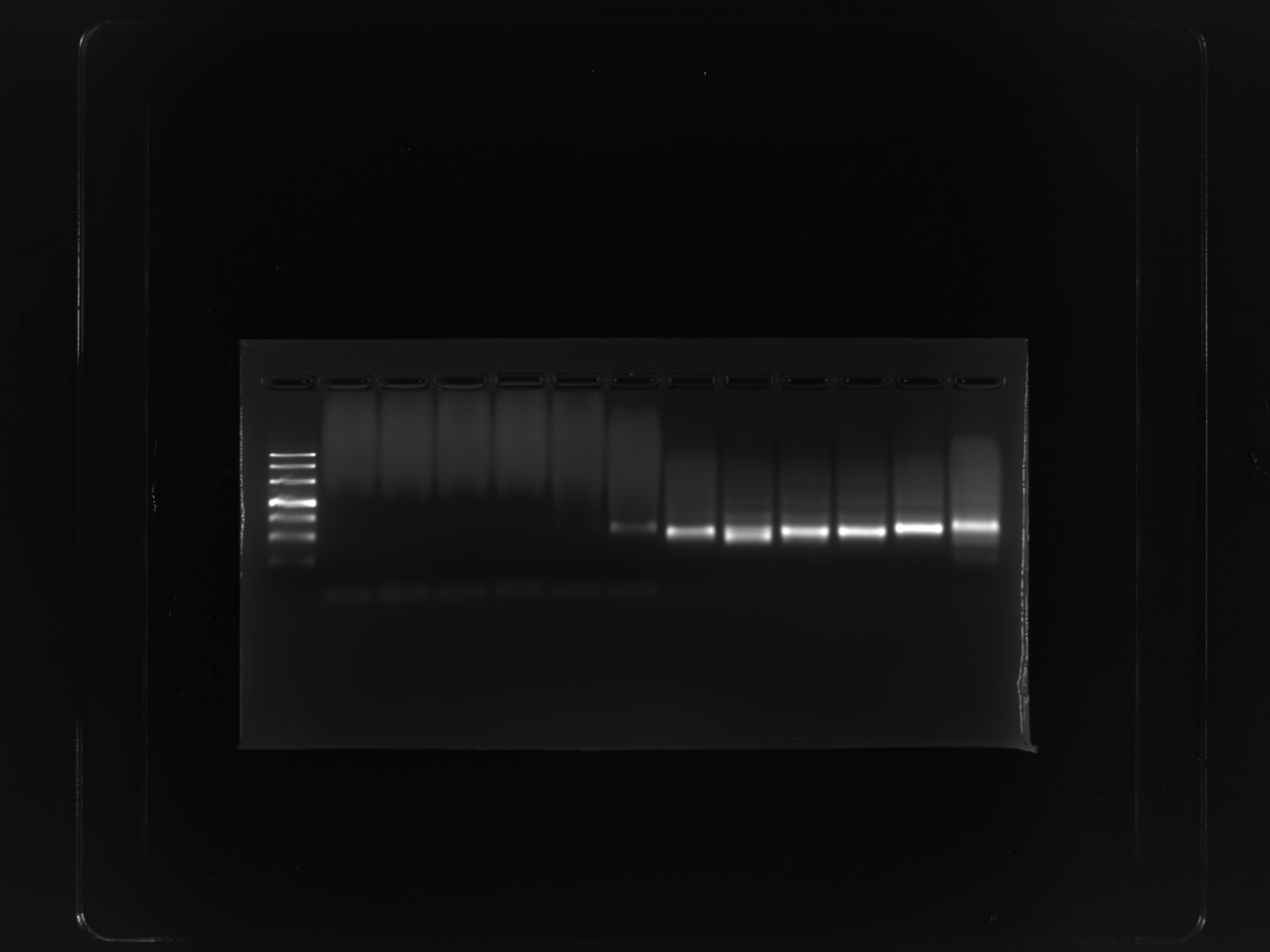

Supplement: Supplementary file 1 [file biosensors-14-00476-s001.zip › Figure S2/5 thú¿1ú¬.tif]

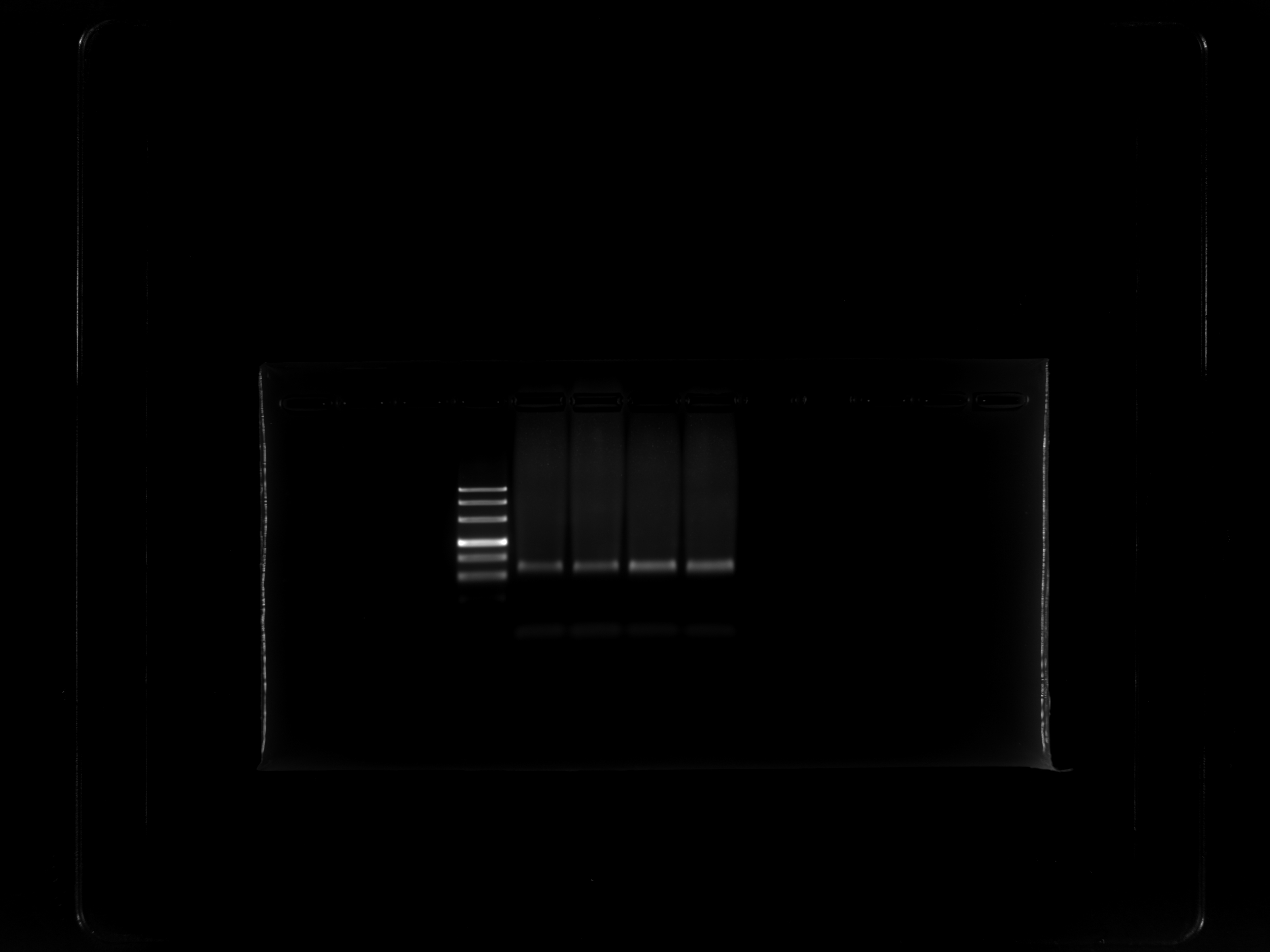

Supplement: Supplementary file 1 [file biosensors-14-00476-s001.zip › Figure S2/5 thú¿2ú¬.jpg]

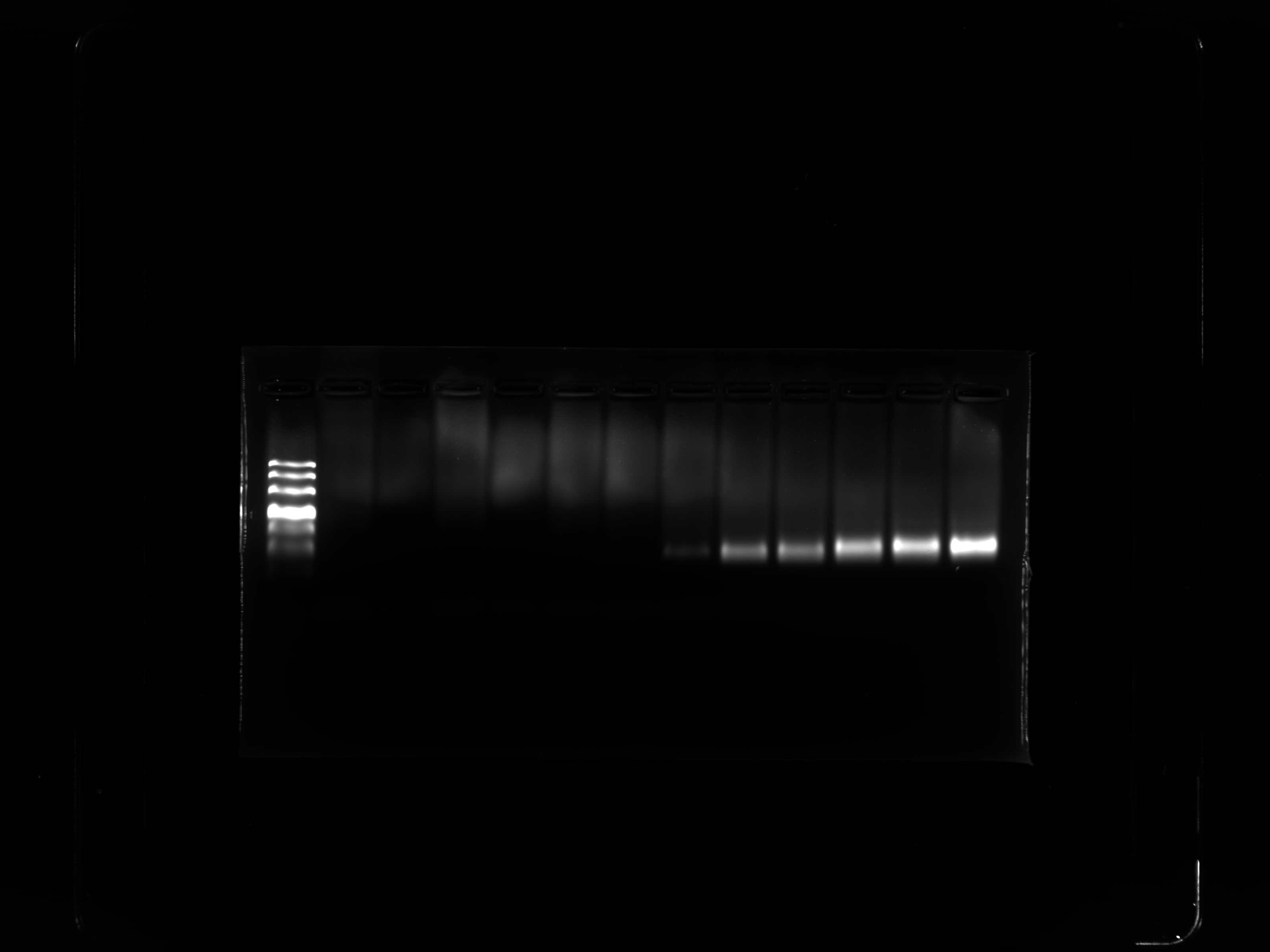

Supplement: Supplementary file 1 [file biosensors-14-00476-s001.zip › Figure S2/6 th (1).jpg]

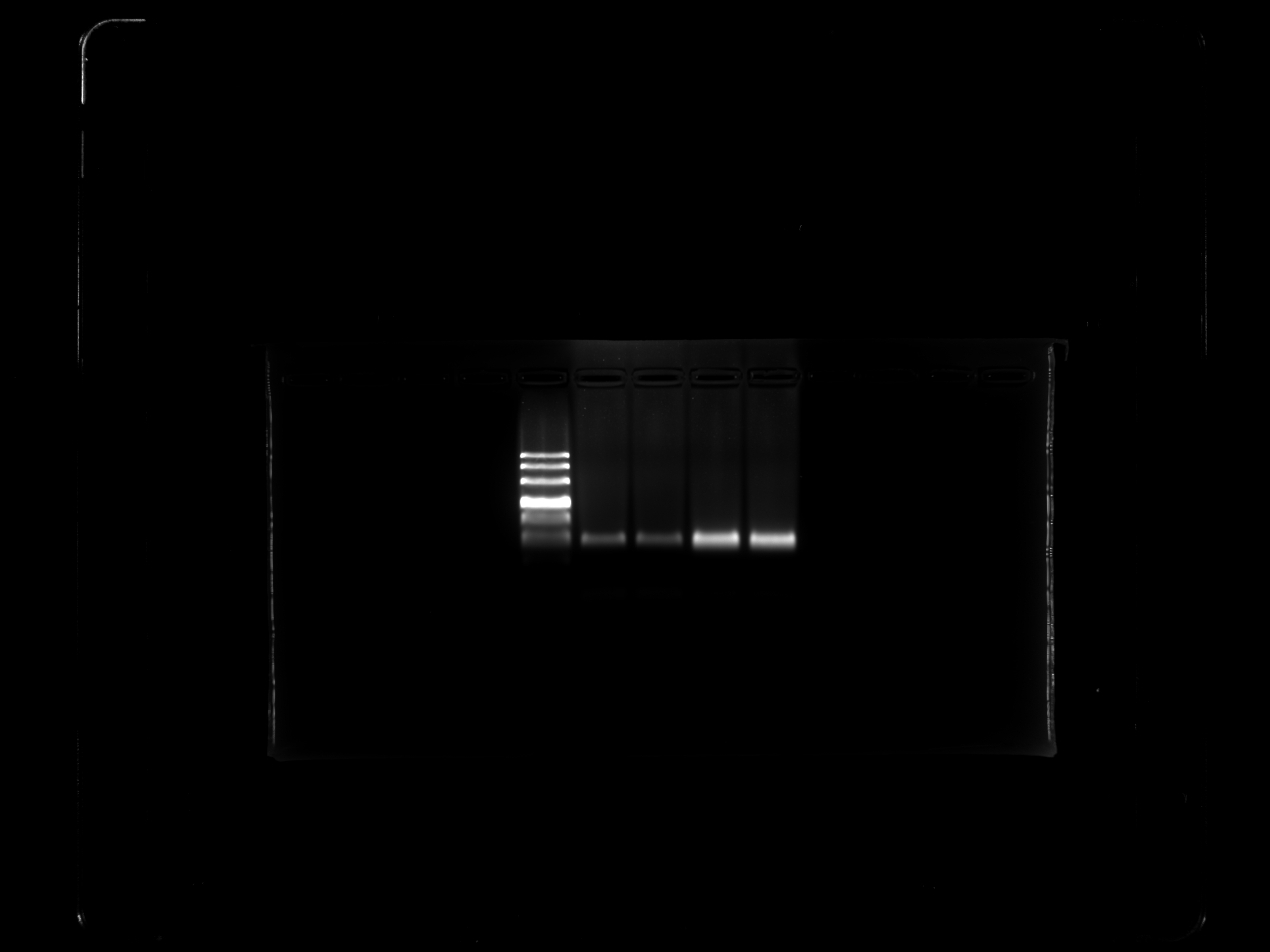

Supplement: Supplementary file 1 [file biosensors-14-00476-s001.zip › Figure S2/6 th (2).jpg]

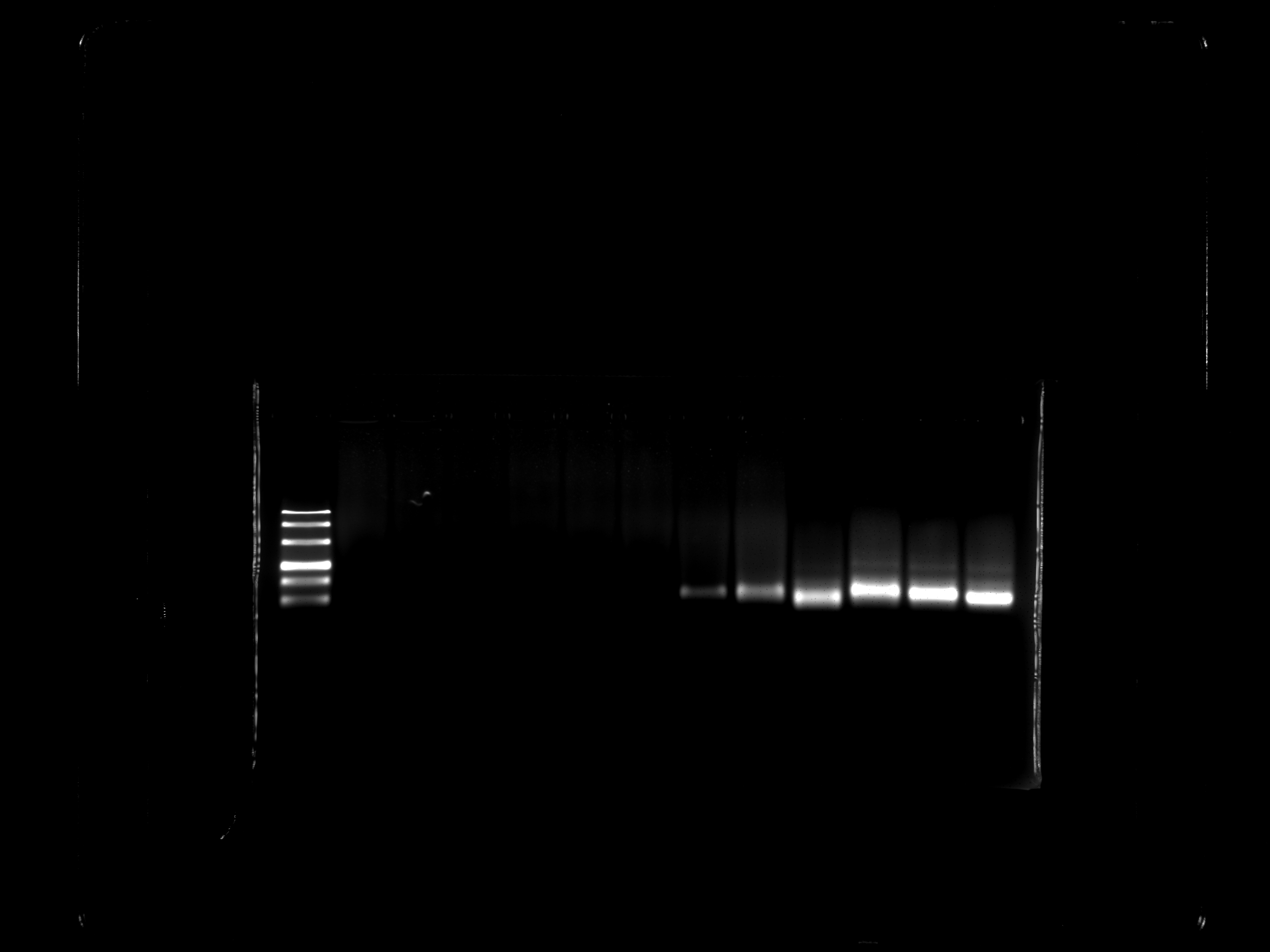

Supplement: Supplementary file 1 [file biosensors-14-00476-s001.zip › Figure S2/7 thú¿1ú¬.jpg]

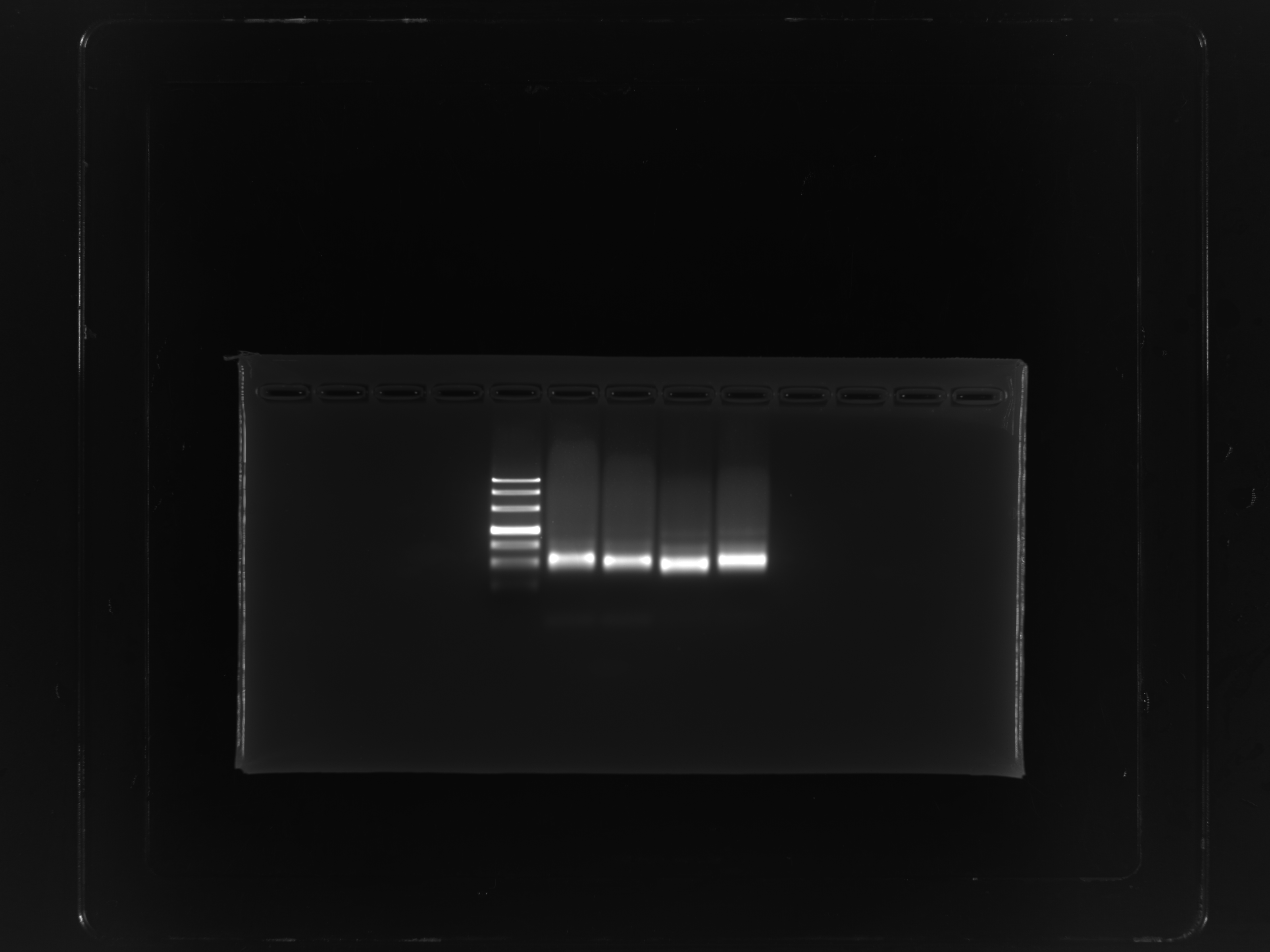

Supplement: Supplementary file 1 [file biosensors-14-00476-s001.zip › Figure S2/7 thú¿2ú¬.tif]

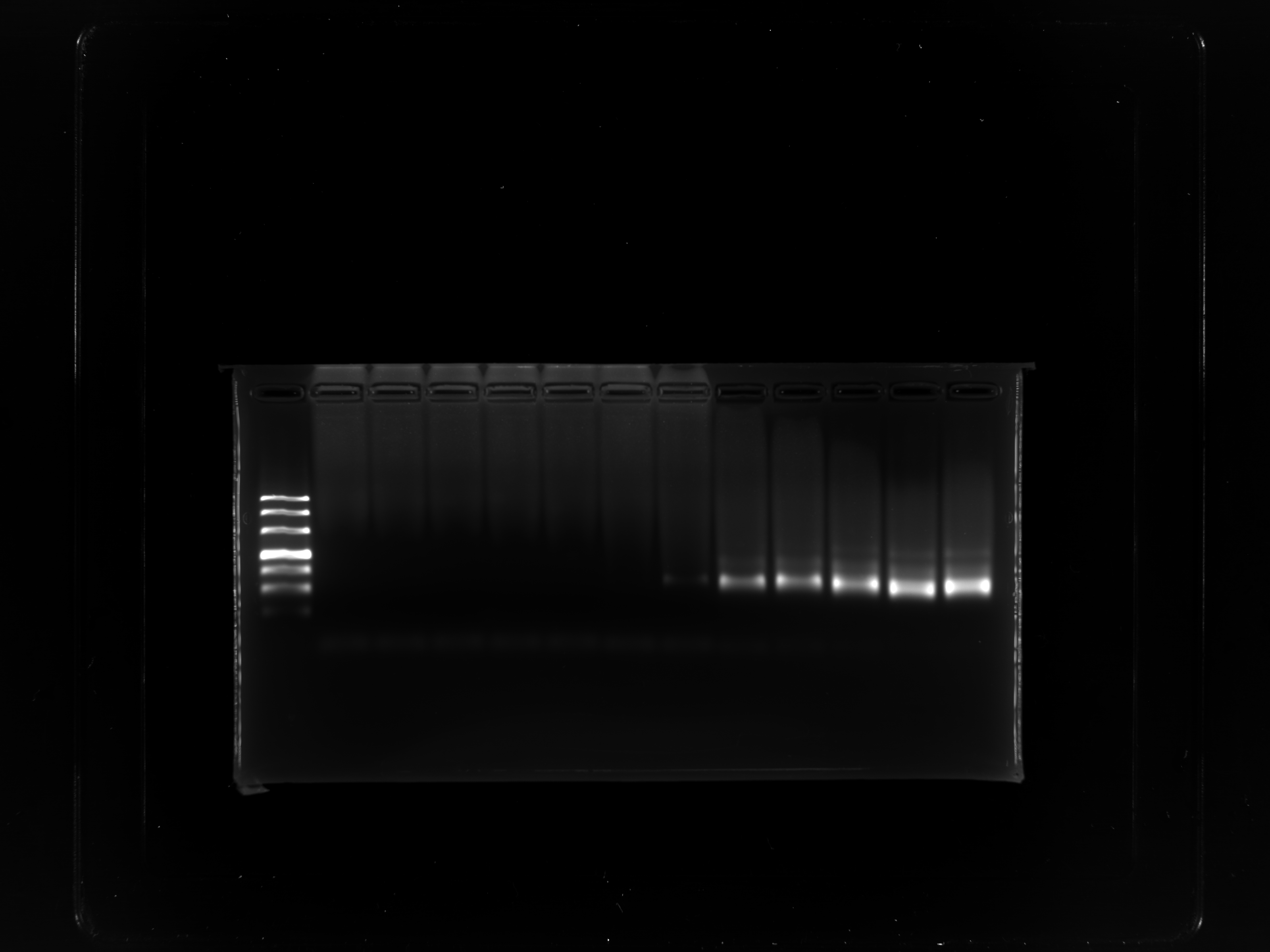

Supplement: Supplementary file 1 [file biosensors-14-00476-s001.zip › Figure S2/8 th (1).jpg]

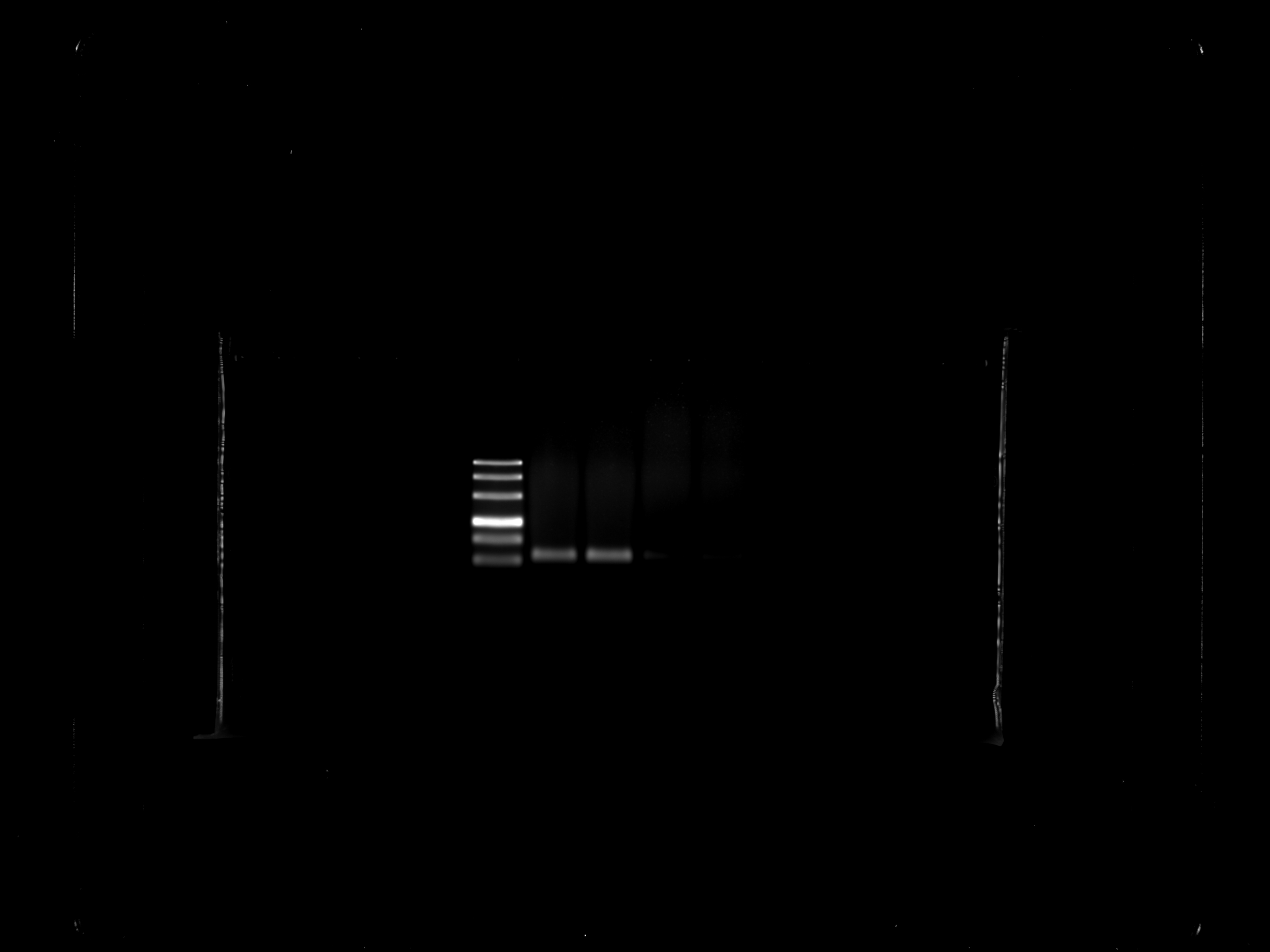

Supplement: Supplementary file 1 [file biosensors-14-00476-s001.zip › Figure S2/8 th (2).jpg]

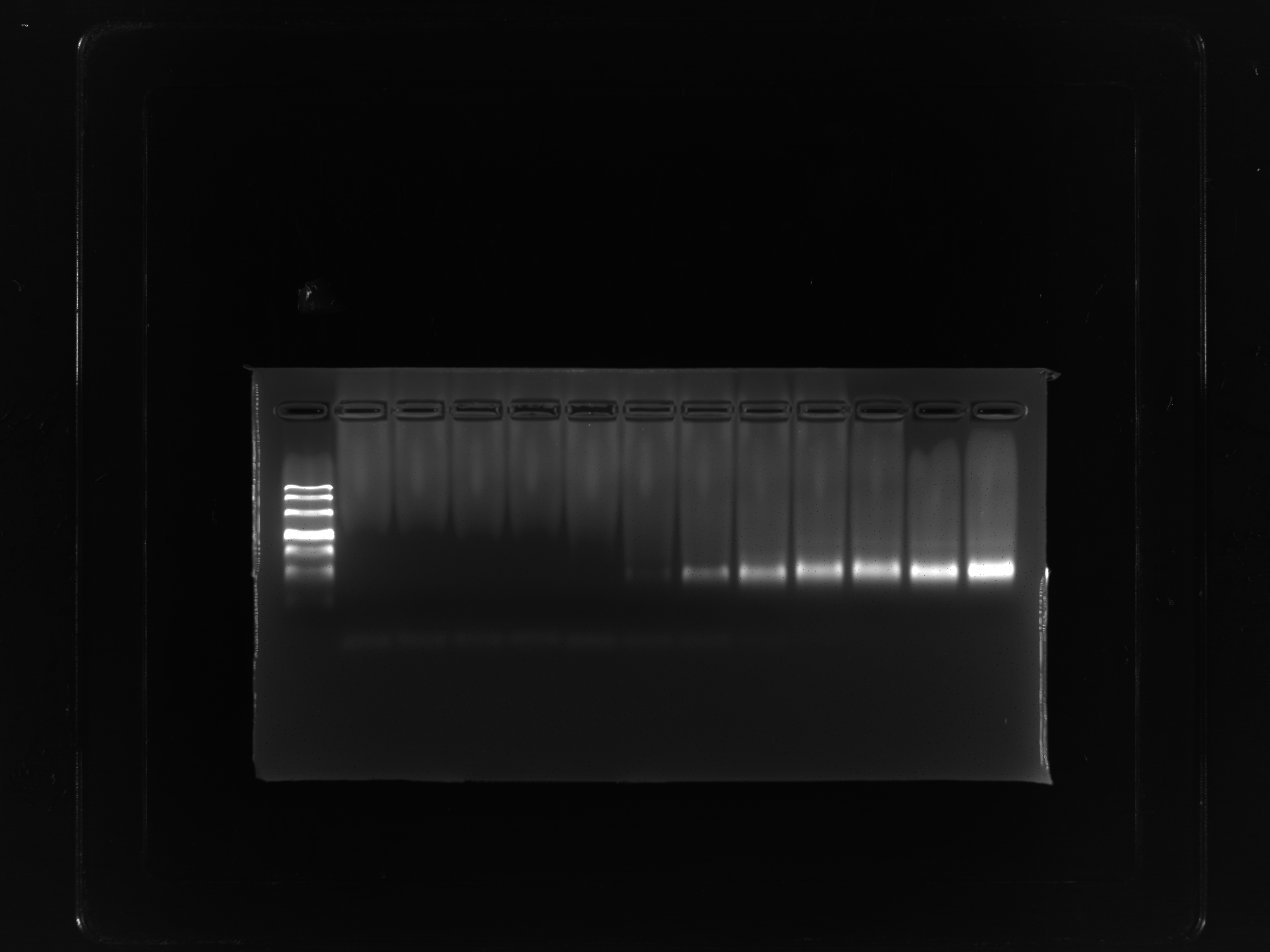

Supplement: Supplementary file 1 [file biosensors-14-00476-s001.zip › Figure S2/9 thú¿1ú¬.tif]

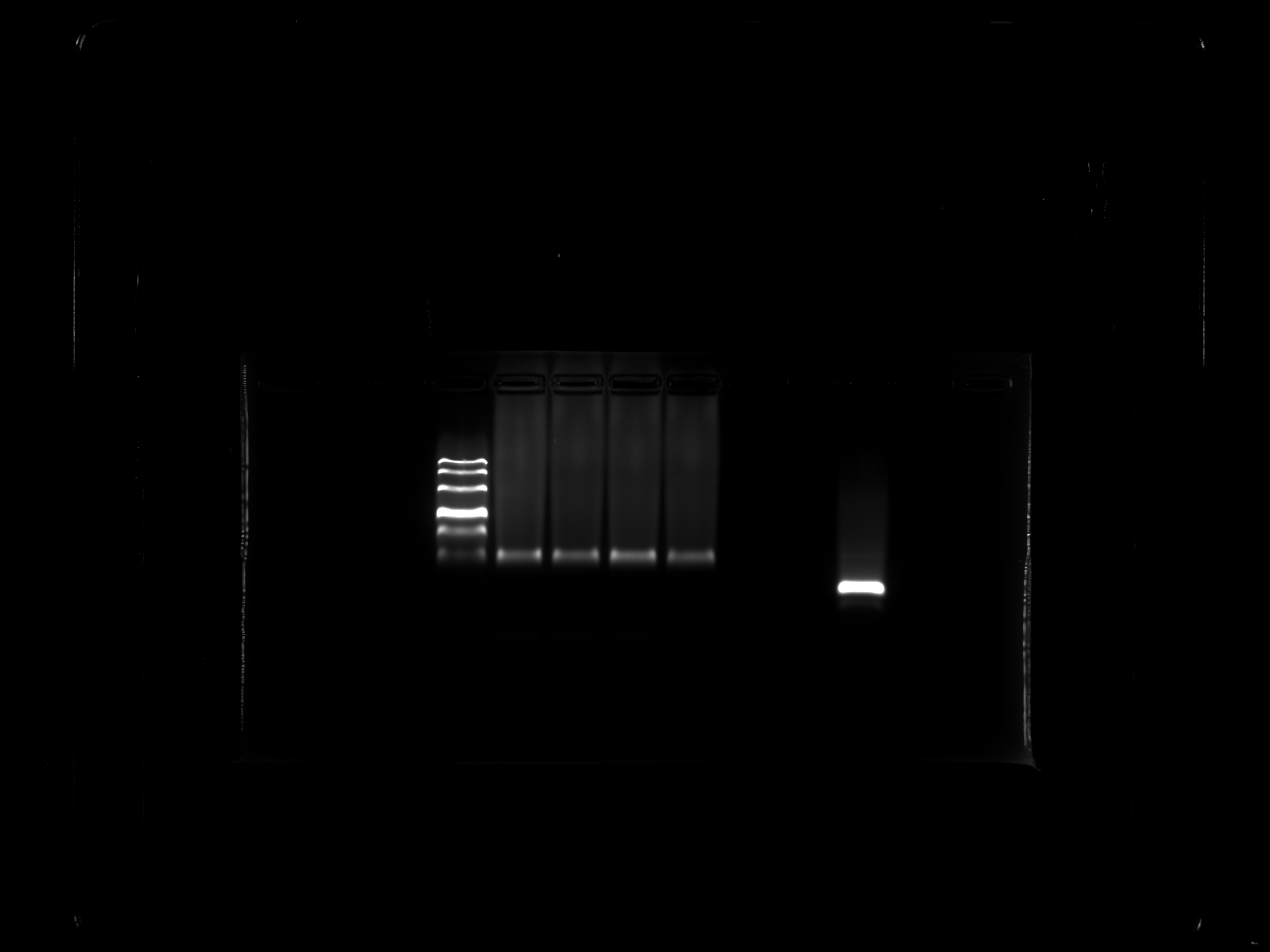

Supplement: Supplementary file 1 [file biosensors-14-00476-s001.zip › Figure S2/9 thú¿2ú¬.jpg]
